# Strategizing AI utilization for psychological literature screening: A comparative analysis of machine learning algorithms and key factors to consider

**DOI:** 10.1017/rsm.2025.10053

**Published:** 2025-12-19

**Authors:** Lars König, Steffen Zitzmann, Martin Hecht

**Affiliations:** 1 Department of Psychology, Helmut-Schmidt-University, Germany; 2 Department of Psychology, MSH Medical School Hamburg, Germany

**Keywords:** AI-aided literature screening, machine learning, meta-analysis, stopping criteria, systematic reviews

## Abstract

With the rapid growth of scholarly literature, efficient artificial intelligence (AI)–aided abstract screening tools are becoming increasingly important. This study evaluated 10 different machine learning (ML) algorithms used in AI-aided screening tools for ordering abstracts according to their estimated relevance. We focused on assessing their performance in terms of the number of abstracts required to screen to achieve a sufficient detection rate of relevant articles. Our evaluation included articles screened with diverse inclusion and exclusion criteria. Crucially, we examined how characteristics of the screening data—such as the proportion of relevant articles, the overall frequency of abstracts, and the amount of training data—impacted algorithm effectiveness. Our findings provide valuable insights for researchers across disciplines, highlighting key factors to consider when selecting an ML algorithm and determining a stopping point for AI-aided screening. Specifically, we observed that the algorithm combining the logistic regression (LR) classifier with the sentence-bidirectional encoder representations from transformers (SBERT) feature extractor outperformed other algorithms, demonstrating both the highest efficiency and the lowest variability in performance. Nonetheless, the algorithm’s performance varied across experimental conditions. Building on these findings, we discuss the results and provide practical recommendations to assist users in the AI-aided screening process.

## Highlights

### What is known?

- The performance of machine learning (ML) algorithms for artificial intelligence (AI)–aided screening has been examined across various research fields. These studies have shown that performance varied between algorithms and across different datasets (collections of abstracts).

### What is new?

- Our study focused on evaluating the performance of 10 ML algorithms used for AI-aided screening.
- We systematically manipulated the prevalence of relevant abstracts, the overall frequency of abstracts, and the amount of training data to examine the impact of these factors on the algorithms’ performance.

### Potential impact for RSM readers

- Readers will gain insights into the effectiveness and robustness of the tested algorithms, assisting them in selecting an algorithm and determining the optimal stopping point for the AI-aided screening. Based on our findings, we offer recommendations that challenge and refine current screening practices, providing guidance on key factors to consider in developing an effective screening strategy.

## 1 Introduction

The integration of advanced technologies such as machine learning (ML), large language models (LLMs), and generative artificial intelligence (AI) has been increasingly acknowledged and adopted within the scientific research community. These technologies serve various purposes, ranging from generating ideas and summarizing articles to create code and analyzing results.^1^ ^,^ ^2^ Their adoption can expedite the research process, thereby contributing to the ongoing acceleration of scientific output.^3^ ^,^ ^4^ For instance, the advancement of generative AI has enabled researchers to create a research paper within a remarkably short time frame of 1 h.^5^ Consequently, methods such as meta-analyses and systematic reviews become increasingly paramount. They serve as indispensable tools for synthesizing research findings,^6^ ^,^ ^7^ identifying robust effects,^8^ assessing the overall quality of the evidence,^9^ ^,^ ^10^ and deriving research policies.^11^ Unfortunately, these methods are often resource intensive, requiring numerous hours of skilled labor.^12^ Thereby, a considerable portion of the workload involves searching and screening for relevant articles.^13^ ^,^ ^14^ However, both tasks can be expedited using modern innovative tools, conserving resources, and improving sustainability.^15^ ^–^ ^17^

**Figure 1:**
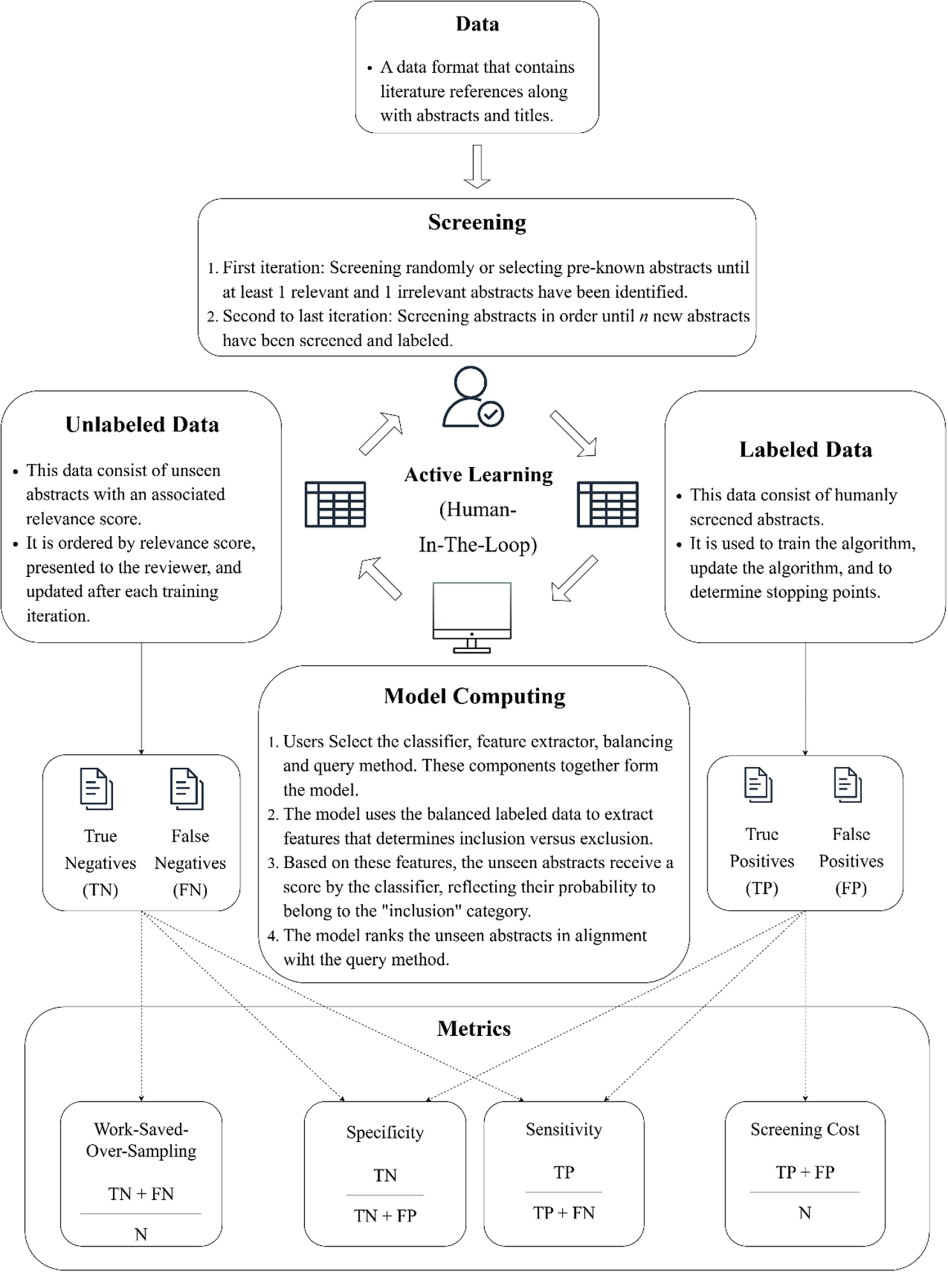
Active learning in the realm of AI-aided screening. *Note*: The human-in-the-loop approach, along with the metrics used to evaluate screening performance.

In pursuit of accelerating the abstract screening, a variety of AI-aided screening tools have been developed.^18^ ^,^ ^19^ These tools predominantly utilize ML to expedite the literature screening process by organizing abstracts based on their anticipated relevance, thereby facilitating the swift discovery of all relevant articles. To achieve this goal while maintaining control over study selection, these tools use an active learning approach—a human-in-the-loop process (Figure 1). The reviewer begins by screening abstracts until at least one relevant example and one irrelevant example have been identified. These labeled abstracts are then used to train an ML algorithm, which ranks the remaining abstracts according to their estimated relevance. The reviewer subsequently screens the top-ranked abstracts, and the newly labeled data are fed back into the training set, enabling the algorithm to refine its predictions in an iterative cycle.

Evaluating the effectiveness of different tools through various studies showed that in 50% of the cases, screening about 40% of the abstracts was sufficient to identify 95% of the relevant articles. However, the studies exhibited considerable variability. In 25% of the studies, only 14% of the abstracts needed to be screened, whereas in the upper 25%, at least 68% of the abstracts had to be screened to achieve the same identification rate.^20^ Among other factors, this variability in performance relied on the ML algorithm used for ordering abstracts and the literature, which was screened. These discrepancies, in turn, affected the performance of stopping criteria designed to aid users in deciding when to stop their AI-aided screening process.^21^

Unfortunately, experimental investigations into factors that influence the performance of ML algorithms used in these tools remain scarce.^18^ ^,^ ^21^ ^,^ ^22^ Consequently, users of these tools often encounter uncertainty regarding when to stop screening while ensuring the identification of the most relevant articles. This concern has been highlighted by researchers who screened all abstracts despite utilizing AI-aided screening tools (e.g., Marsili and Pellegrini^23^). To address the uncertainty inherent in AI-assisted abstract screening, we compared the performance of multiple ML algorithms using a systematically collected, heterogeneous set of abstracts from five distinct domains within psychology, ranging from clinical to educational research. This dataset captures performance across interdisciplinary domains, each with its own specificities, such as differences in inclusion and exclusion criteria and varying degrees of terminological precision, while also reflecting the variability inherent to a broader research field. It thus provides insights into performance differences that may also arise in other disciplines.

We further examined algorithm performance under systematically manipulated characteristics of the abstract collections—specifically, the proportion of relevant abstracts and the overall size of the collection—extending previous correlational findings on contextual influences (e.g., Campos et al.^22^). In addition, we assessed the impact of training set composition to evaluate how different initial conditions affect subsequent performance, offering insights into the role of early-stage decisions in the screening process. While our analyses focus on ASReview, the broad range of ML algorithms tested also yields information relevant for other tools that employ the same algorithms. In addition, the influence of the tested factors informs users of other tools about important aspects to consider such as characteristics of the abstract collections when conducting AI-aided screening.

Taken together, this work provides performance estimates under empirically varied conditions and offers practical guidance for AI-aided screening. Importantly, these estimates can inform the critical decision of when to stop screening, a choice with substantial consequences for identifying relevant literature.

## 2 AI-aided screening

AI-aided screening tools powered by ML algorithms offer a promising solution to accelerate research synthesis.^4^ ^,^ ^18^ ^,^ ^24^ These tools typically employ an *active learning* approach—a human-in-the-loop method in which an ML algorithm determines the screening order and continuously updates its predictions based on the decisions of a human reviewer (see Figure 1). In semi-automatic screening, human verification of the model’s predictions plays a central role, with the verified information fed back into the algorithm for retraining.^19^ The screening order is determined by the similarity between the training set and unseen abstracts, prioritizing those most likely to be relevant. Once new abstracts are labeled, they are added to the training set, enabling the algorithm to update its ranking and refine the selection process in each iteration.^24^ ^,^ ^25^

### 2.1 Machine learning in AI-aided abstract screening

ML algorithms employed in AI-aided screening tools typically involve two primary processes: feature extraction and classification.^25^ ^,^ ^26^ Feature extraction identifies pertinent information, such as phrases, keywords, and patterns, from both the training set (already labeled abstracts) and the screening set (unseen abstracts). Within this study, feature extraction techniques, such as term frequency-inverse document frequency^27^ ^,^ ^28^ (TFIDF), doc2vec,^29^ and sentence-bidirectional encoder representations from transformers^30^ (SBERT), are employed. The extracted information is then transformed into numerical data. Subsequently, using this information, classifiers predict the likelihood for each unseen abstract of belonging to the relevant category.^25^ ^,^ ^26^ In this study, we utilize the random forest^31^ (RF), support vector machine^32^ ^,^ ^33^ (SVM), fully connected neural network with two hidden layers^34^ (nn-2-layer), logistic regression^35^ (LR), and naïve Bayes^36^ ^–^ ^38^ (NB) for this task. The combination of a feature extractor and a classifier is here referred to as an ML algorithm. Note that not every feature extractor is compatible with every classifier. For example, the NB classifier cannot process negative values generated by the Doc2Vec or SBERT feature extractors.

Besides these ML algorithms, in tools such as ASReview, two additional methods can be applied that influence the ranking of abstracts: the query and the balancing strategy.^39^ The query strategy specifies how the algorithm utilizes information for ranking abstracts. For example, the certainty-based query strategy orders unseen abstracts based on their estimated probabilities of inclusion. Other strategies may cluster abstracts by similarity or introduce randomness into the ranking by including a percentage of randomly selected abstracts.^26^ ^,^ ^40^ The balancing strategy modifies the training data utilized by the ML algorithms to prevent the algorithm from becoming overly sensitive to details related to the more prevalent irrelevant abstracts, which could impair its ability to identify relevant abstracts. To counter this, some tools implement rebalancing techniques, such as undersampling irrelevant abstracts while maintaining relevant ones.^39^ ^,^ ^41^

### 2.2 Overview of AI-aided screening research

To highlight the importance of gaining a deeper understanding of the factors influencing ML algorithm performance, we will briefly outline performance metrics, the current research landscape, and how stopping criteria—designed to help users determine when to stop the screening process—are related to the performance of these algorithms.

#### 2.2.1 Performance metrics for machine learning algorithms

Numerous metrics are available to assess the performance of ML algorithms. Many metrics, such as *relevant records found (RFF)* and *work saved over sampling (WSS)*, evaluate performance in terms of *sensitivity*, also referred to as recall.^42^ ^–^ ^44^ To clarify these metrics and the data on which they are based, we provide an overview in Figure 1 (see above). Sensitivity (Eq. 1), for example, is defined as the ratio of correctly identified relevant abstracts or *true positives (TPs)* to the total number of relevant abstracts:
(1)

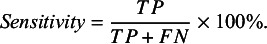



This total number of relevant abstracts encompasses both the *TPs* and the *false negatives (FNs)*, with the latter representing relevant abstracts that were not screened and thus not identified.

The RRF metric reflects sensitivity after screening a certain percentage of all abstracts. Specifically, an RFF after screening 10% (RFF@10%) of 40% indicates that 40% of relevant abstracts were identified after screening 10% of all abstracts. In contrast, the WSS metric indicates the percentage of abstracts that do not require screening at a prespecified sensitivity level.^18^ ^,^ ^22^ ^,^ ^26^ ^,^ ^44^ For instance, a WSS at 95% sensitivity (WSS) of 40% indicates that 95% of the relevant abstracts were identified after screening 60% of the abstracts. Consequently, 40% of the abstracts did not require screening. In the context of AI-aided screening, achieving a 95% sensitivity is generally considered satisfactory.^45^ This threshold is informed by the understanding that traditional random screening is susceptible to misclassifying about 10% of the abstracts due to factors such as fatigue.^46^ Moreover, identifying the final 5% of relevant abstracts can require a disproportionately large increase in screening time when using AI-aided screening tools.^44^ Thus, aiming for 100% identification might reduce the benefit of using these tools. Additionally, research has shown that excluding the last 5% of studies did not impact the outcomes of a meta-analysis.^43^ However, some performance measures, such as the *average time to discovery*, do not require arbitrary cutoff values such as the 95% threshold.^26^ Similarly, the *area under the curve (AUC)* offers valuable insights into algorithmic performance, as it integrates sensitivity and specificity (see Khalil et al.^42^).

However, despite this advantage, these measures lack an intuitive interpretation that would guide users of AI-aided screening tools on when to stop screening. To provide these users with this information, we measured performance as the percentage of abstracts that required screening in order to achieve a sensitivity of 95%. Therefore, we express performance as *screening cost (SC)*. The SC metric represents the ratio of abstracts that have been screened, including both TP and false positive (FP), to the total number of abstracts ( 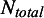 ):
(2)

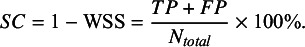



Thus, the SC reflects the opposite of the WSS. It informs users about the percentage of abstracts they need to screen to achieve a certain level of sensitivity. For instance, an SC of 40% indicates that 95% of the relevant articles were identified after screening the abstracts of 40% of the articles retrieved from the literature search.

#### 2.2.2 Performance of AI-aided screening

A recent review on the performance of AI-aided screening tools reported an average SC of approximately 50% to achieve 95% sensitivity, highlighting their potential to expedite systematic reviews by halving the workload. However, considerable variability in performance was noted both within and between tools. One contributing factor to this variability was the ML algorithm employed.^20^ Comparing performance for different ML algorithms within the same AI-aided screening tool (i.e., ASReview) revealed differences in SC among algorithms of around 10%, with the combination of the LR classifier and the SBERT feature extractor (LR + SBERT) outperforming others including the RF + SBERT algorithm.^22^ ^,^ ^43^ Accordingly, the number of abstracts that need to be screened can differ by about 10% depending on the algorithm. For example, in a collection of 1,000 abstracts, the best-performing algorithm may reduce the workload by roughly 100 abstracts compared to the lowest-performing one. This underscores the impact that the choice of algorithm has on the effectiveness of AI-aided screening tools, which, in turn, impacts the performance of criteria used to decide when to stop the screening process.^22^

Another crucial factor influencing the performance of AI-aided screening tools is the nature of the text data, specifically the abstracts identified through the literature search.^18^ ^,^ ^22^ For instance, a recent study evaluated the performance of ASReview for different medical-related abstract collections. In this study, 2% to 70% of the abstracts required screening to identify 95% of the relevant articles.^47^ The complexities of different research domains, especially when dealing with more intricate inclusion criteria, might account for observed discrepancies in the performance of AI-aided screening tools.^22^ Additionally, previous research has shown that the performance of an ML algorithm in ranking abstracts by relevance can be influenced by the researcher conducting the screening, underscoring the importance of how these criteria are applied.^47^

Beyond the research domain, both the prevalence of relevant abstracts and the volume of abstracts emerged as impacting the effectiveness of ML algorithms. Indeed, a recent study demonstrated that the relative performance of these algorithms improved when the datasets included more abstracts. In contrast, a higher prevalence of relevant abstracts was associated with decreased algorithm performance.^22^ However, this decline may be attributed to the relative increase in relevant abstracts, which naturally extends screening time due to the greater number of relevant articles in the dataset. Moreover, because these findings are based on unmanipulated datasets, further systematic evaluations are needed before drawing firm conclusions.

Lastly, the performance of ML algorithms is influenced by the composition of the training set used to create the initial ranking of abstracts. Users must pretrain algorithms with domain-specific vocabulary or other relevant data to enhance contextual understanding and improve the ability to distinguish between relevant and irrelevant abstracts.^42^ Most AI-aided screening tools, including ASReview used in this study, require at least one relevant abstract and one irrelevant abstract to generate an initial ranking based on predicted relevance. Typically, algorithm performance improves early in the screening process, because more abstracts resembling the training set are identified. In contrast, performance tends to decline toward the end, as relevant abstracts that are less similar to the training set are less likely to be assigned a high probability of relevance. Ultimately, algorithm performance depends on the initial training set, which influences which abstracts are subsequently added to the training data.^44^ ^,^ ^47^ Additionally, the number of relevant abstracts in the training set has been shown to affect ML algorithm performance, with larger training sets generally leading to better outcomes. However, these effects have been inconsistent across different datasets.^20^ ^,^ ^48^ Some authors also argued that abstracts from articles known to be relevant before conducting a literature search might bias the algorithm’s performance, when used as an initial training set.^45^ According to the authors, these abstracts might share specific similarities that could lead to overfitting, limiting the algorithm’s ability to detect abstracts that do not share these similarities despite being relevant. To mitigate this bias, the authors recommended randomly selecting a training set that includes at least one relevant abstract and one irrelevant abstract. Following this reasoning, increasing the number of randomly selected abstracts could mitigate the influence of specific characteristics of the training set, potentially enhancing algorithm performance and reducing variability arising from the use of different training sets. When this assumption holds, using five randomly selected abstracts for training should result in lower variability in performance across training sets compared to using only one abstract. In conclusion, AI-aided screening tools hold the promise of substantially reducing screening time. However, given the complex interplay of factors that influence algorithm performance, deriving robust conclusions about their effectiveness necessitates more systematic investigations.

In consideration of these findings, we argue that ML algorithms should be compared by systematically varying factors such as the frequency of relevant and irrelevant abstracts, the prevalence of relevant abstracts, or the training set. An evaluation on the impact of these factors could inform the development of tailored recommendations for the field and increase confidence in the use of these tools.

#### 2.2.3 Stopping AI-aided screening

As noted above, numerous factors influence the theoretical performance of the ML algorithms. In practice, however, performance largely depends on the rules used to determine when to stop screening. Stopping too early can lead to a suboptimal identification rate, whereas stopping too late results in unnecessary screening effort, when performance is constant. Nonetheless, the effectiveness of a stopping rule interacts with algorithm performance and is therefore also influenced by factors affecting algorithm efficiency. When algorithm performance is poor, the stopping rule may be triggered too early; conversely, the same rule may be triggered too late when performance is high. Thus, gaining a deeper understanding of the factors that influence ML algorithm performance, such as the composition of the training set, the prevalence of relevant abstracts, and the size of the abstract collection, is crucial for selecting appropriate stopping rules and adapting them effectively. This argument is supported by findings from other fields, where correlations of these factors and algorithm performance have been observed in unmanipulated data.^22^

Several techniques have been developed to assist users in deciding when to stop screening. For instance, heuristic stopping techniques can be applied directly during the screening process, regardless of the tool used. Their ease of integration makes them particularly popular among users, especially when working with ASReview, as noted by König et al.^21^ Notably, despite their simplicity, these heuristics yielded promising results. For example, the *data-driven heuristic* determines the stopping point based on a predefined number of consecutive irrelevant abstracts—commonly defined as 50 in a row.^49^ As a result, this heuristic is sensitive to the order in which abstracts are presented, which can vary depending on the ML algorithm used.^43^

Similarly, the time-based heuristic determines the stopping point as a predefined percentage of abstracts screened. Its effectiveness depends on whether the algorithm has identified all relevant abstracts before this threshold is reached.^50^ For example, when screening stops after 30% of the total abstracts have been reviewed, sensitivity would be 50% if only half of the relevant abstracts were identified within that 30%. Thus, this heuristic is directly influenced by the algorithm’s ability to rank abstracts effectively, requiring its cutoff value to be adjusted accordingly. Indeed, the performance of both the heuristic and the algorithm’s ranking accuracy has been shown to depend on the specific cutoff values used,^51^ ^–^ ^54^ as well as the number of studies in the training set.^55^ Moreover, performance varied considerably depending on the ML algorithm applied, the literature being screened, and characteristics of the collection, such as the prevalence of relevant abstracts.^21^

It is interesting to note that combining the data-driven and time-based heuristics has been shown to outperform the individual application of each rule in terms of efficiency.^22^ This finding supports previous recommendations to integrate multiple stopping criteria. For example, the SAFE method for AI-aided screening, developed through expert consensus, combines several stopping criteria across four screening phases: screen a random set for training data (S), apply active learning (A), find more relevant records with a different model (F), evaluate quality (E).^45^

The first screening phase involves randomly screening a subset of the abstracts identified by the literature search. This phase can be stopped once at least 1% of the total amount of abstracts has been screened and at least one relevant abstract has been identified. This initial random prescreening enables the identification of abstracts that serve both as training data for the algorithm and as key studies. Key studies are those known to be relevant before initiating AI-assisted screening but are intentionally embedded within the pool of unscreened abstracts. These key studies can serve as a stopping criterion, requiring the algorithm to present them to the reviewer before the screening process can be stopped. This approach ensures that screening continues until all preidentified key studies have been retrieved, thereby potentially enhancing the sensitivity of the AI-aided screening process. An additional benefit of the initial random screening phase is its capacity to randomly sample abstracts for training the ML algorithm. Studies identified as relevant prior to the literature search may share features that are not central to the research question but could be disproportionately weighted by the algorithm. By introducing a broader range of characteristics into the training data through random selection, this approach may help mitigate such bias and reduce the algorithm’s reliance on specific patterns.^45^

The second phase of the SAFE method employs active learning, using abstracts identified during the first phase to train the ML algorithm. In this phase, four stopping criteria must be met before screening can be stopped: (1) All key studies must be identified; (2) at least twice the number of relevant abstracts found during the first (random screening) phase must be discovered; (3) at least 10% of all abstracts must be screened (time-based heuristic); and (4) 50 consecutive abstracts must be labeled as irrelevant (data-driven heuristic).

However, the cutoff values for the time-based and data-driven heuristics as used by the SAFE method were found to perform poorly in a recent study. Achieving a sensitivity of approximately 95% required screening at least 30% of the abstracts and stopping only after 5% of the abstracts in a row were deemed irrelevant.^22^ Furthermore, the effectiveness of these cutoff values varied across algorithms and was influenced by both the total number of abstracts and the proportion of relevant abstracts. These findings highlight the importance of gaining a deeper understanding of factors such as the proportion of relevant abstracts, abstract collection size, and training data characteristics in order to select and adapt appropriate stopping rules.

## 3 The present study

As outlined above, existing literature on the performance of ML algorithms and the stopping criteria dependent on them has produced mixed findings. Moreover, there is a noticeable gap in systematic investigations into the factors influencing the performance of ML algorithms for AI-aided screening and, consequently, the performance of stopping criteria. Therefore, experimental studies on factors such as the composition of the screening data (e.g., the proportion of relevant abstracts and the total abstract collection size) and the influence of the training set, which the ML algorithm uses to create the initial screening order, could provide valuable insights for users of these tools.

The dataset used in this study, originally compiled by König et al.,^21^ comprises systematically collected abstracts from five distinct domains within psychology (e.g., clinical psychology and educational psychology), with multiple journals represented within each domain. Although limited to psychology, these domains encompass diverse research topics and exhibit overlap with other scientific disciplines. We summarized the research topics examined in the original meta-analyses in Supplementary Table S1. Using systematically collected data from within a single research field provides a valuable complement to studies (e.g., Ferdinands et al.^26^) evaluating tool performance across different scientific disciplines. Likewise, controlling for broader disciplinary influences offers a counterpoint to findings from more diverse datasets, where performance variations have been shown to be substantial, e.g., Harmsen et al.^47^

In contrast to previous research, we manipulate key factors related to the composition of relevant and irrelevant abstracts as well as the configuration of training sets. Specifically, the factors examined in this study—namely, the prevalence of relevant abstracts, the size of the abstract collection, and the number of training studies—are likely to have broader applicability, offering insights for optimizing AI-assisted screening across diverse scientific disciplines. For example, Campos et al.^22^ studied the performance of ML algorithms in education and educational psychology but did not manipulate dataset characteristics, even though they observed correlations between these factors and performance. Our findings may therefore help researchers in such fields align cutoff values for time-based heuristics more closely with the characteristics of their data, ultimately improving both the efficiency and effectiveness of AI-aided screening.

Although the present work is mainly focused on algorithms within the AI-aided screening tool ASReview, some of these algorithms are also employed in other tools. For example, the most widely used classifier—the SVM—is examined here when paired with the TF-IDF feature extractor. Our approach involved two simulation studies designed to comprehensively evaluate algorithm performance under varied conditions. We employed 10 different ML algorithms (i.e., LR + doc2vec, LR + SBERT, LR + TFIDF, NB + TFIDF, nn2layer + doc2vec, nn2layer + SBERT, RF + doc2vec, RF + SBERT, RF + TFIDF, and SVM + TFIDF) to compare their performance. By assessing the performance using SC at a sensitivity of 95%, our findings provide critical insights for users of AI-aided screening tools, particularly regarding the choice of cutoff value for the time-based heuristic stopping technique.

The first study evaluated the algorithms’ performance across 21 abstract collections with systematically varied prevalence ratios of relevant abstracts (i.e., 0.5%, 1%, 5%, and 10%) and when trained with different sets of only one relevant abstract and one irrelevant abstract. Within this study, we aimed to answer the following research questions:

**RQ1.1:** How do the 10 ML algorithms perform in terms of SC across different prevalence conditions?

**RQ1.2:** How does the performance (SC) of the 10 ML algorithms vary across the abstract collections, prevalences of relevant abstracts, and varying training sets?

The first research question (RQ1.1) aimed to provide ASReview’s users with an estimate of the percentage of abstracts that need to be screened to identify 95% of the relevant literature. This estimate considers various use cases, including different ML algorithms and abstract collections with varying prevalences of relevant abstracts. Moreover, it can guide users when selecting cutoff values for the time-based heuristic stopping criterion. To offer users additional insights, the second research question (RQ1.2) sought to provide information on the robustness of these estimates. Given that researchers cannot predict whether the algorithm will perform well or poorly for their specific abstract collection and training set, providing a range of performance estimates can help adjust the estimate more conservatively if needed.

The second study delved deeper into factors that might influence the performance of ML algorithms, such as the number of abstracts used to train the algorithm, the frequency of abstracts (abstract collection size), and the prevalence of relevant abstracts, with prevalence being manipulated differently compared to Study 1. To this end, we measured performance for the best-performing algorithm from Study 1 trained with either 1, 2, or 5 relevant and 10 irrelevant abstracts. Additionally, we established two abstract collection size conditions, with maximum frequencies of 2,000 and 4,000 abstracts, respectively, and adjusted the prevalence of relevant abstracts to 1%, 2.5%, and 5%. In contrast to Study 1, we kept the number of relevant abstracts constant across different prevalence conditions to deepen our understanding of the prevalence effect. This approach aimed to clarify the following research questions:

**RQ2.1:** How does prevalence influence the performance (SC) of the ML algorithm when the number of relevant abstracts is held constant across prevalence conditions?

**RQ2.2:** How does abstract collection size influence the performance (SC) of the ML algorithm when prevalence is held constant?

**RQ2.3a:** Does increasing the number of relevant abstracts in the training set enhance the algorithm’s performance (SC)?

**RQ2.3b:** Does increasing the number of relevant abstracts in the training set reduce variability in performance (SC) across AI-aided screening simulations using different training sets?

In this study, we held the number of relevant abstracts constant across different prevalence conditions, allowing us to isolate the effect of prevalence without the confounding influence of varying numbers of relevant abstracts on the algorithm. A clearer understanding of the impact of prevalence can lead to more precise performance estimates, helping users determine the minimum percentage of abstracts to screen (RQ2.1). Similarly, examining the effect of abstract collection size could provide valuable insights for users regarding how to adjust these estimates (RQ2.2). In addition, exploring the impact of varying numbers of relevant abstracts in the training set could help users determine whether identifying more than one relevant abstract before initializing AI-aided screening increases the performance of the algorithm. As the abstracts in the training set establish the initial ranking for screening, using a greater number of relevant abstracts to train the ML algorithm might prevent overfitting to the specific characteristics of the training set. This could result in shorter screening times due to improved algorithm performance (RQ2.3a). Furthermore, by reducing the impact of specific characteristics in the training set, variability in performance across different training sets might be minimized. This could enhance the accuracy of performance estimates and improve the robustness of recommendations for stopping AI-aided screening (RQ2.3b).

## 4 Study 1

### 4.1 Method

In Study 1, we reexamined data from König et al.,^21^ which was generated through simulations of AI-aided screening using the screening tool ASReview. While the authors focused on the performance of stopping criteria for AI-aided screening, our focus was on the performance of the algorithms. Below, we briefly describe the process by which the authors sourced collections of abstracts that had been screened for meta-analyses in the field of psychology. Additionally, we outline the methods used to modify the prevalence of relevant abstracts within these collections, as well as the simulation procedures employed for the AI-aided screening process. We then summarize our approach for reusing these data in evaluating the performance of ML algorithms.

This study’s design and analysis were not preregistered. All code and results are openly shared on Open Science Framework (OSF; https://osf.io/53ter/). The simulated data used to evaluate ML algorithm performance were taken from König et al.,^21^ except for the RF + SBERT condition, which we simulated separately to include this additional promising algorithm. Although these data are not published due to their large size, they will be made available upon request. The abstract collections on which this simulation is based can be retrieved from König et al.’s^21^ OSF repository (https://osf.io/7yhrq) under the Creative Commons Attribution 4.0 International (CC BY 4.0) license.

### 4.2 Data

In their study, König et al.^21^ identified previously published meta-analyses in psychology and requested the corresponding reference lists, including the screened abstracts and their inclusion decisions. To be eligible for their simulation study on stopping rules in AI-aided screening tools, a meta-analysis had to involve the screening of at least 1,000 abstracts, with a minimum of 50 abstracts labeled as relevant based on abstract screening. Systematic reviews without a meta-analysis were excluded.

Additionally, the authors searched for meta-analyses across 6 different psychological research domains and within 17 journals per domain. This approach aimed to minimize dependency on domain-specific characteristics and mitigate potential similarities arising from journal guidelines. To select journals, the authors ranked them using the Journal Citation Indicator (JCI) from the *Journal Citation Reports*,^56^ which accounts for contextual relevance and enables comparisons across research domains. Their initial goal was to request data from 180 meta-analyses, with 30 per domain, evenly distributed across journals. From the initial pool of 180 eligible meta-analyses, 21 datasets were obtained from the psychological domains of applied psychology (*n* = 4), clinical psychology (*n* = 3), developmental psychology (*n* = 5), educational psychology (*n* = 5), and social psychology (*n* = 4). As shown in Supplementary Table S1, the meta-analyses also varied in their aims and research topics.

A unique feature of the simulation data created by König et al.^21^ is the manipulation of the prevalence of relevant abstracts. To adjust the proportion of relevant abstracts within a collection of abstracts, the authors randomly sampled relevant and irrelevant abstracts from the original collection until prevalence ratios of 0.5%, 1%, 5%, and 10% were achieved. Thereby, the original prevalence ratio was either increased by reducing the number of irrelevant abstracts or decreased by reducing the number of relevant abstracts. For instance, to achieve a prevalence ratio of 5% in an abstract collection with a true prevalence ratio of 4%, they excluded irrelevant abstracts. Conversely, in a collection with a true prevalence ratio of 6%, they excluded relevant abstracts until a prevalence of 5% was met. This procedure was repeated 1000 times for each abstract collection and prevalence ratio condition, resulting in 84,000 *artificial abstract collections*. All abstract collections and the frequencies of relevant and irrelevant abstracts are displayed in Table 1.

**Table 1:** Descriptives of the original and artificially constructed abstract collections (Study 1)

	Original abstract						
	collection			Artificial abstract collection			
				*N;* 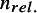			
				Meta-analysis	R	*N;* 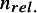	Ratio	0.5%	1%	5%	10%
Alden et al. (2021)^63^	A	1486; 59	4.13	1206; 6	1313; 13	1176; 56	616; 56
Bottema-Beutel et al. (2021)^71^*	D	6283; 743	13.41	5226; 26	5252; 52	5523; 263	5786; 526
Bourke et al. (2023)^72^*	D	9308; 158	1.73	8643; 43	8686; 86	3150; 150	1650; 150
Castro-Alonso et al. (2021)^73^	E	2351; 217	10.17	2010; 10	2020; 20	2121; 101	2222; 202
Dailey and Bergelson (2022)^74^*	D	4992; 203	4.24	4422; 22	4545; 45	4032; 192	2112; 192
Endendijk et al. (2020)^75^	S	1271; 274	27.48	804; 4	909; 9	987; 47	1034; 94
Estevez Cores et al. (2021)^76^	A	1784; 227	14.58	1407; 7	1414; 14	1533; 73	1617; 147
Hall et al. (2023)^77^*	E	13531; 544	4.19	12261; 61	12423; 123	10836; 516	5676; 516
Hsieh et al. (2022)^78^	S	2343; 107	4.79	2010; 10	2121; 21	2121; 101	1111; 101
Karabinski et al. (2021)^64^	A	1840; 70	3.95	1608; 8	1616; 16	1386; 66	726; 66
Khazanov et al. (2022)^79^	C	3423; 250	7.88	3015; 15	3030; 30	3150; 150	2607; 237
Leijten et al. (2021)^65^*	C	4382; 262	6.36	3819; 19	3939; 39	4095; 195	2728; 248
Liu et al. (2020)^61^	C	1585; 579	57.55	804; 4	909; 9	987; 47	1045; 95
Ober et al. (2020)^80^*	E	6124; 718	13.28	5025; 25	5151; 51	5376; 256	5643; 513
Reimer and Sengupta (2023)^81^	S	2661; 219	8.97	2211; 11	2323; 23	2415; 115	2288; 208
Schindler et al. (2023)^82^	S	2272; 414	22.28	1608; 8	1717; 17	1848; 88	1936; 176
Simonsmeier et al. (2022)^83^*	E	9768; 1507	18.24	7839; 39	7878; 78	8232; 392	8624; 784
Tang et al. (2022)^84^	E	2053; 53	2.65	1809; 9	1919; 19	1050; 50	550; 50
Vermillet et al. (2022)^85^	D	1875; 206	12.34	1407; 7	1515; 15	1659; 79	1738; 158
Woods et al. (2022)^86^*	A	5955; 265	4.66	5427; 27	5454; 54	5271; 251	2761; 251
Zaneva et al. (2022)^60^*	D	7266; 89	1.24	6834; 34	6868; 68	1764; 84	924; 84

*Note:* This table is adapted from König et al.^21^ * = used in Study 2; *N* = total number of abstracts; 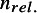 = number of relevant abstracts; R = research domain; Ratio = prevalence ratio; D = developmental; C = clinical; S = social; E = educational; A = applied psychology.

### 4.3 Simulation

Utilizing the 84,000 manipulated abstract collections, König et al.^21^ simulated AI-assisted abstract screening for each dataset. Thereby, the authors employed ASReview,^44^ an open-source AI-assisted screening tool that supports various ML algorithms. Specifically, they used ASReview’s built-in simulation mode, which leverages prelabeled abstracts to mimic a reviewer’s decision-making process. This mode can be accessed via a user interface, the command line, or the Python Application Programming Interface (API).^39^ To conduct a simulation, a dataset containing abstracts and their corresponding inclusion decisions is required. ASReview then estimates the relevance of each abstract based on a training set and generates a screening order. The system evaluates the highest-ranked abstract based on the stored inclusion decision and continues this process iteratively. Thus, simulation mode does not make independent assumptions about abstract relevance. The mode simply mirrors how a researcher would likely progress in screening.

Beyond the training set, simulation mode also allows users to specify the ML algorithm, query strategy, and balancing method. In their simulations, König et al.^21^ used ASReview’s default settings for querying and balancing: the certainty-based query strategy and the dynamic resampling balancing method. The certainty-based strategy ranks abstracts based on predicted relevance, while the dynamic resampling method mitigates the risk of oversampling irrelevant abstracts by undersampling irrelevant ones and oversampling relevant ones while maintaining a balanced training dataset. Although ASReview offers alternative querying and balancing methods, these settings are considered among the most suitable for AI-aided abstract screening.^26^

Furthermore, König et al.^21^ employed nine ML algorithms: LR + doc2vec, LR + SBERT, LR + TFIDF, NB + TFIDF, nn2layer + doc2vec, nn2layer + SBERT, RF + doc2vec, RF + TFIDF, and SVM + TFIDF. To this set, we added the RF + SBERT algorithm, following a reviewer’s suggestion, as this combination has shown strong performance in previous studies (e.g., Campos et al.^22^).

As for screening with ASReview, in simulation mode, users select the training set either randomly or manually by choosing specific studies. In their study, König et al.^21^ initialized each simulation run with one relevant abstract and one irrelevant abstract as the training set. To ensure consistency across 1,000 replications, they set a seed when using the Python API. This approach resulted in varying training sets across replication runs while maintaining similar training sets across ML algorithms.

A unique feature of simulation mode is the ability to adjust the number of abstracts screened before the model is retrained. When screening abstracts with ASReview, the inclusion probabilities are usually recalculated after each newly screened abstract. However, for this simulation, this parameter was set to 10 in order to improve computational efficiency and save computational resources and time. Thus, the ranking of abstracts was updated after every 10th instead of every labeled abstract.

Using these settings, we generated 84,000 manipulated abstract collections, each screened with all 10 ML algorithms, resulting in a total of 840,000 simulations. Each simulation produced an abstract collection with the order in which abstracts were screened, which we used to calculate algorithm performance. Further details on the methodology and findings of König et al.^21^ can be found in their article and supplementary materials on OSF (https://osf.io/7yhrq). The code and materials for the present simulations are available in our OSF repository (https://osf.io/53ter/).

### 4.4 Performance measures

In contrast to the focus of König et al.,^21^ the present study aimed to evaluate the performance of ML algorithms. Thereby, performance was measured as SC (see Eq. 2). Consistent with prior research on ML algorithms, we evaluated performance at a 95% sensitivity level. For example, an SC of 30% indicates that 95% of the relevant literature is identified after screening 30% of all abstracts. As higher prevalences of relevant abstracts inherently necessitate screening more abstracts, even when algorithms perfectly rank unseen abstracts, we also measured the *FP rate (FPR)*: 
(3)

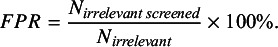



This metric quantifies the percentage of irrelevant abstracts screened relative to the total number of irrelevant abstracts. Thus, it captures the differences in performance across various prevalence conditions while accounting for the varying numbers of relevant abstracts. We measured the FPR at a sensitivity of 95% (FPR).

### 4.5 Analysis

To give users guidance on potential stopping points when using these tools, we assessed the performance using SC and reported several statistical measures: mean, standard deviation, median, interquartile range (IQR), and 90th percentile. These metrics can help users select a cutoff value for the time-based heuristic that aligns with empirical estimates. Given that the data are not normally distributed, we focused on the median, IQR, and 90th percentile in our report. While the IQR reflects variability, the 90th percentile is reported as a conservative performance estimate. We visualized these percentiles through bar plots, delineating the main effects of the ML algorithm, the prevalence ratio, and their interaction. The primary effect of the ML algorithm provides users with critical insights into its efficiency and robustness. The interaction between the ML algorithm and the prevalence ratio might assist users in selecting the most efficient ML algorithm for specific prevalences of relevant abstracts. To further explore the distribution of performance estimates, we visualized this distribution separately for each abstract collection using violin plots. All analyses and visualizations were conducted using the statistical software R^57^ employing the packages *dplyr* ^58^ and *ggplot2.* ^59^

### 4.6 Results

**Figure 2:**
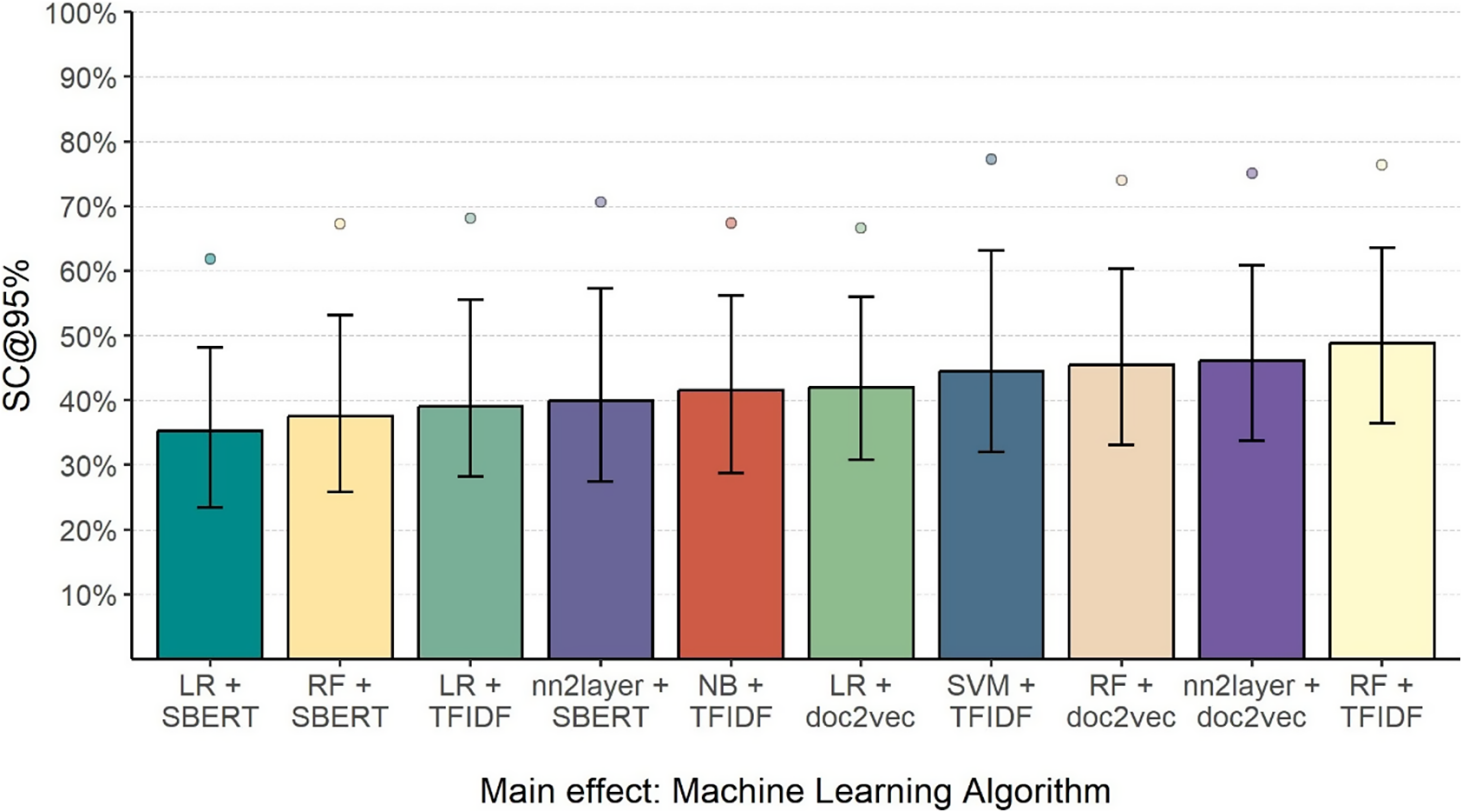
Screening cost at 95% sensitivity by machine learning algorithm (Study 1). *Note*: The bars reflect the median performance, the whiskers represent the interquartile range, and the points represent the 90% percentile. For each ML algorithm, descriptive statistics are based on 84,000 artificial abstract collections. SC@95% represents the percentage of abstracts that needed to be screened to identify 95% of the relevant articles.

As illustrated in Figure 2, the performance of ML algorithms showed considerable variability in regard to the SC. Notably, the LR + SBERT algorithm achieved the best performance (SC = 35.28% [23.42, 48.16]), followed by RF + SBERT (SC = 37.50% [25.86%, 53.12]). In contrast, the RF + TFIDF algorithm showed the worst performance (SC = 48.85% [36.48, 63.60]). The performance of the other algorithms ranged between these. The exact values displayed in Figure 2 are documented in Supplementary Table S2.

**Figure 3:**
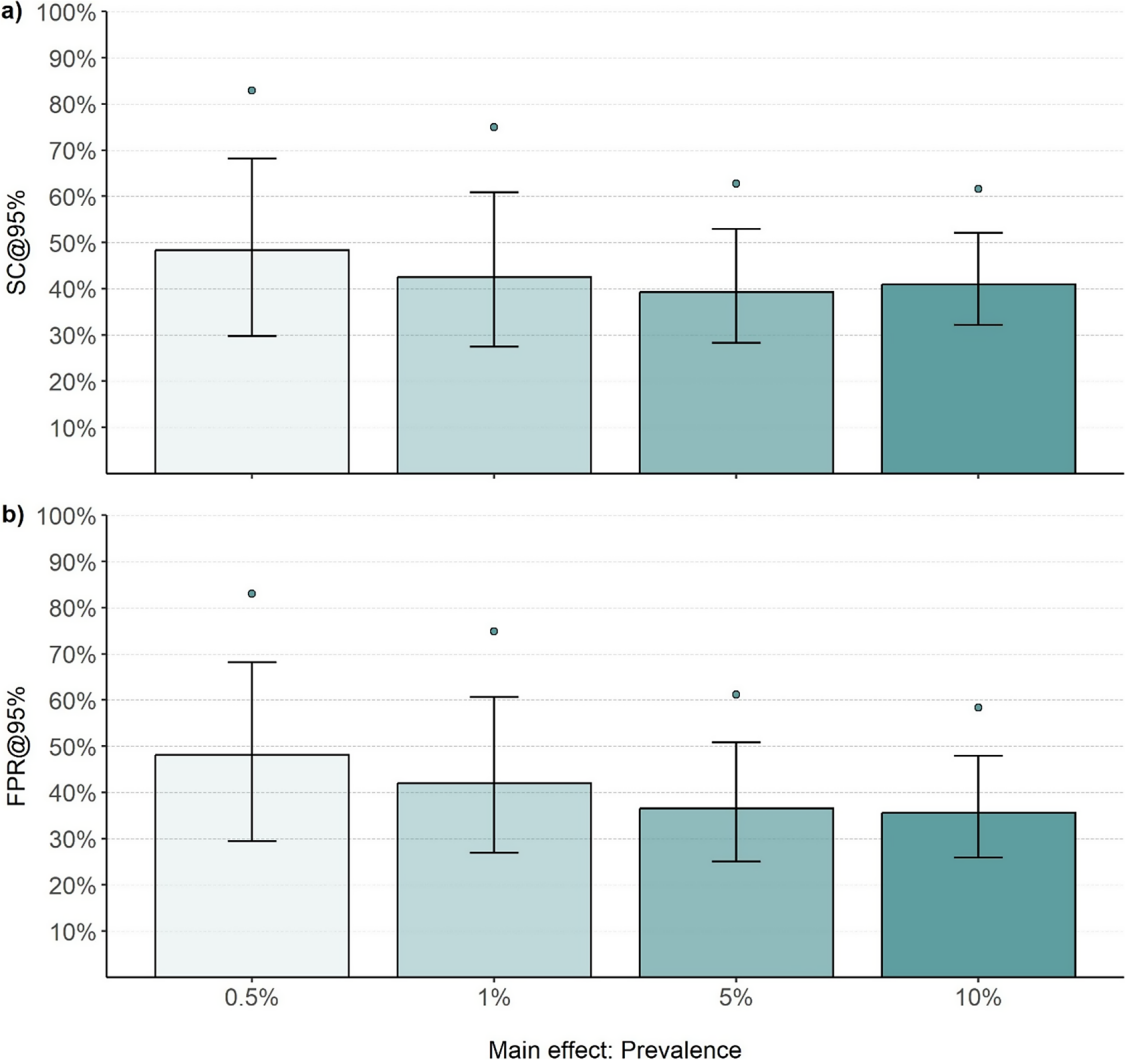
Screening cost and false-positive rate at 95% sensitivity by prevalence (Study 1). *Note*: The bar plots reflect the median performance, the error bars represent the interquartile range, and the points represent the 90% percentile. Each summary statistic summarizes 180,000 observations. a) Performance in terms of Screening Cost (SC), b) performance in terms of False Positive Rate (FPR) when identifying 95% of the relevant literature (@95%).

Analyzing SC across prevalence ratios showed that the average performance of ML algorithms improved as the prevalence of relevant abstracts increased (Figure 3a). The highest median SC was observed in the 0.5% ratio condition (SC = 48.33% [29.75, 68.23]). At 1% (SC = 42.45% [27.50, 60.88] and 5% (SC = 39.23% [28.28, 52.89]) prevalences, both SC and variability decreased. In contrast, the median SC rose slightly again in the 10% prevalence ratio condition (SC = 40.92% [32.12, 52.07]). However, the 90% percentile displayed a consistent improvement with increasing prevalence due to less variability in performance. The specific values shown in Figure 3a can be found in Supplementary Table S3. In addition, comparing the FPR revealed that the percentage of irrelevant abstracts which were screened constantly reduced with an increasing prevalence ratio (Figure 3b).

**Figure 4:**
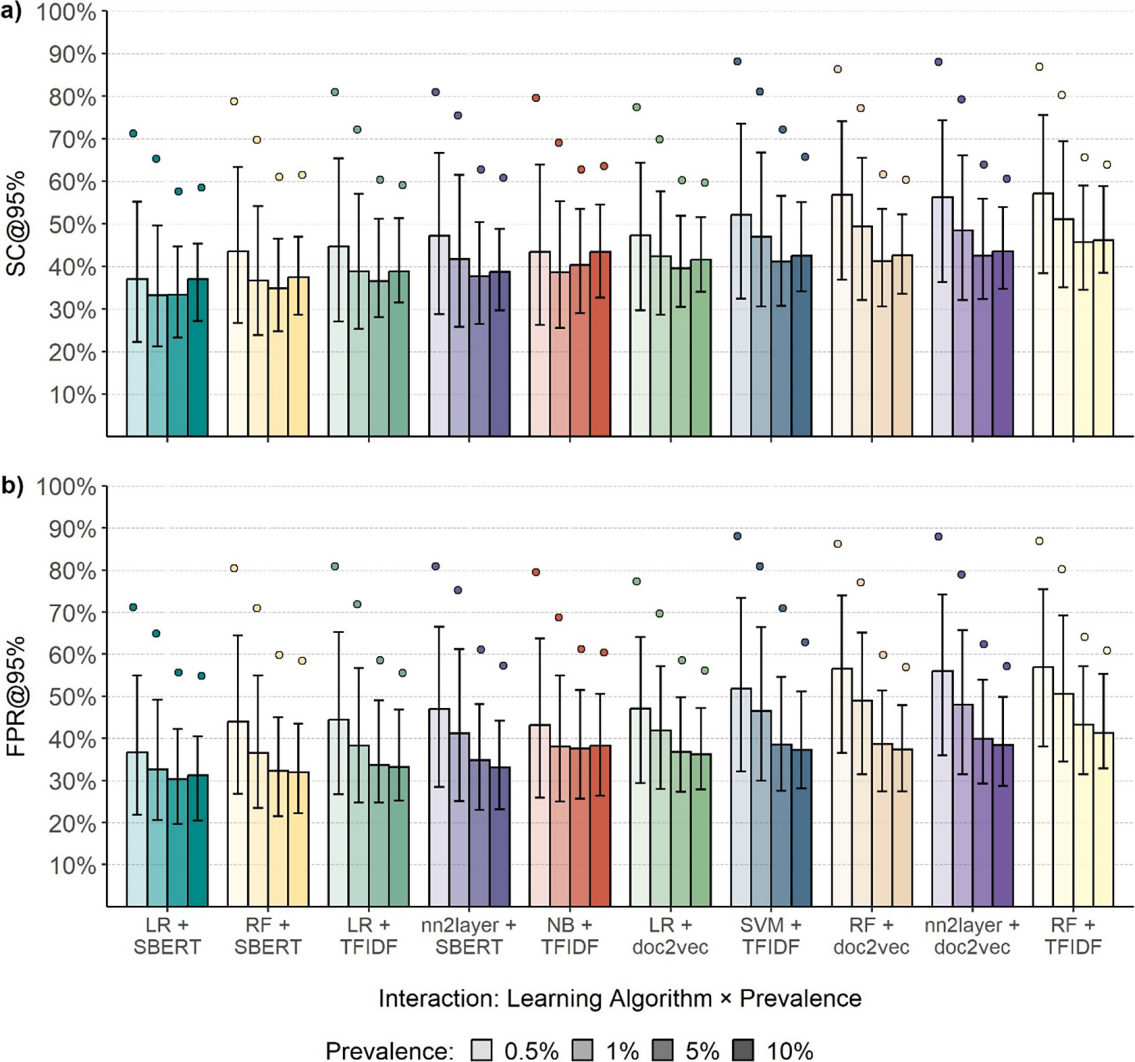
Screening cost and false-positive rate at 95% sensitivity by machine learning algorithm and prevalence (Study 1). *Note*: The bar plots reflect the median performance, the error bars represent the interquartile range, and the points reflect the 90% percentile. Each summary statistic summarizes 21,000 observations. a) Performance in terms of Screening Cost (SC), b) performance in terms of False Positive Rate (FPR) when identifying 95% of the relevant literature (@95%).

The interaction between the factors’ prevalence ratio and the ML algorithm showed that differences among algorithms diminished as prevalence increased (Figure 4a). Similarly, the variability within each algorithm decreased as prevalence rose. The LR + SBERT algorithm consistently outperformed the other algorithms in all prevalence ratio conditions (see Table 2). The ranking of other algorithms, however, slightly fluctuated based on the prevalence ratio. For instance, the NB + TFIDF algorithm ranked as second in the 0.5% condition but sixth in the 10% condition (Table 2). In addition, whereas the median performance of the LR + SBERT algorithm varied only by 4% across different prevalence ratios, the median performance of other algorithms, such as RF + doc2vec, varied by 15%. A surprising observation is the lower performance of the algorithms in the 10% prevalence ratio condition compared to the 5% condition. However, comparing the FPR (Figure 4b) showed that the percentage of irrelevant abstracts requiring screening consistently decreased with an increase in prevalence, except for the LR + SBERT and NB + TFIDF algorithms. For these algorithms, performance, measured as FPR, decreased as the prevalence ratio increased from 5% to 10%.

**Table 2:** Screening cost at 95% sensitivity by ML algorithm and prevalence ratio (Study 1)

ML algorithm	Prevalence	*M*	*SD*	*Mdn*	*IQR*	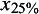	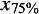	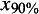
LR + SBERT	0.5%	39.30	22.45	36.97	32.95	22.19	55.14	71.23
		1%	35.92	20.33	33.20	28.35	21.24	49.59	65.27
		5%	34.76	16.03	33.39	21.44	23.23	44.67	57.56
		10%	37.15	14.16	37.02	18.23	27.16	45.39	58.50
LR + TFIDF	0.5%	46.57	24.28	44.64	38.43	26.99	65.42	80.93
		1%	41.60	21.46	38.81	31.65	25.36	57.01	72.14
		5%	39.00	15.53	36.55	23.13	28.03	51.17	60.33
		10%	40.62	13.38	38.77	19.77	31.50	51.26	59.12
nn2layer + SBERT	0.5%	47.51	24.59	47.18	37.90	28.78	66.68	80.93
		1%	43.43	23.07	41.64	35.73	25.78	61.52	75.41
		5%	38.66	17.09	37.72	23.90	26.45	50.36	62.72
		10%	39.50	14.81	38.66	19.09	29.67	48.76	60.75
NB + TFIDF	0.5%	45.44	23.93	43.38	37.70	26.19	63.89	79.59
		1%	40.81	20.20	38.56	29.70	25.60	55.29	69.02
		5%	41.50	15.64	40.36	24.58	28.94	53.52	62.77
		10%	43.67	14.24	43.41	21.93	32.62	54.55	63.57
LR + doc2vec	0.5%	47.74	21.90	47.26	34.65	29.64	64.28	77.44
		1%	43.57	19.54	42.43	28.94	28.62	57.57	69.86
		5%	40.43	14.93	39.55	21.34	30.50	51.84	60.26
		10%	42.04	12.76	41.57	17.54	33.98	51.52	59.64
RF + doc2vec	0.5%	55.33	23.44	56.78	37.23	36.86	74.09	86.28
		1%	48.95	21.48	49.35	33.41	32.08	65.49	77.19
		5%	41.43	15.55	41.27	22.89	30.59	53.48	61.54
		10%	42.44	13.21	42.65	18.63	33.56	52.19	60.32
SVM + TFIDF	0.5%	52.56	25.56	52.04	40.99	32.46	73.45	88.13
		1%	48.42	23.46	46.91	36.21	30.54	66.75	81.04
		5%	44.05	18.27	41.15	25.84	30.66	56.51	72.08
		10%	44.32	14.68	42.44	21.02	34.09	55.11	65.72
nn2layer + doc2vec	0.5%	55.29	24.04	56.18	38.01	36.26	74.27	88.04
		1%	49.08	22.17	48.40	33.91	32.09	66.01	79.16
		5%	43.14	16.19	42.50	23.56	32.29	55.85	63.89
		10%	43.42	13.20	43.53	19.23	34.70	53.93	60.59
RF + TFIDF	0.5%	56.23	23.34	57.15	37.13	38.40	75.53	86.91
		1%	51.64	21.66	51.05	34.35	35.06	69.41	80.30
		5%	45.76	15.69	45.68	24.54	34.43	58.97	65.55
		10%	46.94	13.17	46.15	20.38	38.50	58.87	63.91
RF + SBERT	0.5%	44.99	24.20	43.55	36.59	26.71	63.30	78.81
		1%	39.09	21.38	36.67	30.34	23.79	54.13	69.70
		5%	36.28	16.88	34.85	21.69	24.75	46.44	61.07
		10%	38.41	14.97	37.46	18.36	28.61	46.97	61.48

*Note:* Each data point consists of 21,000 observations. In the table cells, SC is represented as a percentage, yet the percent sign is omitted for clarity in presentation. 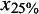 = 25% percentile; 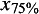 = 75% percentile; 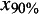 *=* 90% percentile.

An exploratory evaluation of algorithm performance across and within abstract collections yielded several noteworthy observations (see Supplementary Figure S1). Algorithm performance varied substantially within abstract collections due to differences in randomly sampled abstract sets, a consequence of the manipulation design by König et al.^21^ Nonetheless, also in the 10% prevalence condition, where most relevant abstracts were included in all sets (see Table 1), variability was observed. For instance, when applying the LR + SBERT algorithm, the average IQR within abstract collections was 9.79%. In contrast, the average IQR in the 10% prevalence condition was 3%.

The variability between abstract collections, however, remained high despite standardization across abstract collections. In some cases, screening 20% of abstracts was sufficient to identify 95% of the relevant literature (e.g., Zaneva et al.^60^), whereas in others, approximately 65% of abstracts had to be screened to reach the same identification rate (e.g., Liu et al.^61^). Furthermore, performance varied across and within research domains. The best performance was observed for applied psychology (SC = 30.66% [13.88, 44.19]), followed by developmental psychology (SC = 38.11% [27.19, 45.88]), social psychology (SC = 39.82% [29.70, 52.33]), educational psychology (SC = 51.08% [35.36, 62.35]), and clinical psychology (SC = 60.18% [39.92, 69.21]).

### 4.7 Discussion of the results

Consistent with prior research, our first study demonstrated superior performance of the LR + SBERT algorithm.^22^ ^,^ ^43^ This algorithm exhibited the lowest median SC and the least variability in performance across all prevalence ratios. Identifying 95% of the relevant literature required screening between 33% and 37% of the total abstracts, depending on the prevalence of relevant abstracts (RQ1.1).

Interestingly, while the SC of the LR + SBERT and some other algorithms improved as prevalence increased from 0.5% to 1%, it declined at higher prevalence levels. However, due to the prevalence manipulation in the study by König et al.,^21^ higher prevalence conditions included more relevant abstracts, potentially contributing to this observation. Comparing the FPR (see Eq. 3), which reflects the percentage of irrelevant abstracts screened out of all irrelevant abstracts, showed that increased prevalence consistently led to improved performance. Thus, although the total percentage of abstracts to screen increased in higher prevalence conditions, most algorithms performed better in these conditions (RQ1.2). Interestingly, Campos et al.^22^ reported a similar FPR of 35% with abstracts from the field of education and educational psychology. However, their average SC was considerably higher than observed in this study. A possible explanation is that the authors did not standardize prevalence across abstract collections, with some studies showing prevalence above 50% and others below 5%. Nevertheless, the relative stability of the FPRs suggests that this measure may offer more reliable guidance for determining a stopping point than SC, highlighting an intriguing avenue for future research.

A notable limitation of the simulation design from König et al.^21^ is the confounding of the frequencies of relevant and irrelevant abstracts with prevalence. In higher prevalence conditions, the larger number of relevant abstracts led to less variation in the composition of the artificial abstract collection. Thus, the greater number of relevant abstracts in higher prevalence conditions might have contributed to the decreased variability across simulation runs. For example, sampling 20 out of 100 relevant abstracts can yield completely different sets of relevant abstracts, whereas sampling 80 out of 100 consistently results in overlapping sets. Another implication of this finding is that the training set has significantly less influence on performance than the specific abstracts used, even when originating from the same literature search. Nonetheless, even in the 10% prevalence condition, variability across simulation runs emerged, primarily due to differences in the training set. However, the variability in performance both within and between abstract collections highlighted the importance of being mindful of the potential fluctuations in algorithm performance. Users of AI-aided screening tools cannot predict whether their abstract collections or the abstracts used for training the algorithm will result in optimal or suboptimal algorithm performance. Therefore, further research is needed to focus on reducing the variability of algorithm performance in relation to both the training datasets and the abstract collections.

## 5 Study 2

To investigate factors that influence the performance of ML algorithms, we extended the simulation design from Study 1 by (a) standardizing the number of relevant abstracts across different abstract collections and prevalence conditions, (b) creating two conditions that varied in abstract collection size by increasing the frequency of both relevant and irrelevant abstracts, and (c) varying the number of relevant abstracts within the training set. This design allowed for a more controlled examination of how these factors impact algorithm performance. In this study, we did not conduct a comparison of multiple ML algorithms. Instead, we focused on the best-performing algorithm identified in Study 1, the LR + SBERT algorithm. Below, we outline the adjustments made to the simulation design in Study 1 and how these modifications contribute to a deeper understanding of the performance of ML algorithms.

All code and results from this study are openly available on OSF (https://osf.io/53ter/), with the exception of the simulated data, which are not published due to their large size but can be provided upon request. As in Study 1, this research is based on abstract collections originally compiled by König et al.,^21^ which are available under the Creative Commons Attribution 4.0 International (CC BY 4.0) license and can be accessed on OSF (https://osf.io/7yhrq). However, unlike in the first study, we generated the simulated data ourselves by adapting the code of König et al.^21^ The study design and analysis were not preregistered.

### 5.1 Method

In Study 1, we observed performance variations of the ML algorithms across abstract collections, simulation runs, and prevalence conditions. Variability across abstract collections might have arisen from content-related differences and characteristics of the collections, such as the frequency of abstracts included in an abstract collection.^22^ Furthermore, due to the prevalence manipulation by König et al.,^21^ both the number of relevant and irrelevant abstracts varied across prevalence conditions. Notably, larger differences appeared in the number of relevant abstracts compared to irrelevant abstracts, which remained relatively consistent across the different prevalence conditions (see Table 1). Nonetheless, this design limited our ability to attribute variations in the performance of ML algorithms, both between prevalence conditions and across abstract collections, to differences in abstract collection composition (e.g., frequency of relevant and irrelevant abstracts). Therefore, in this study, we standardized the number of relevant and irrelevant abstracts across abstract collections. Additionally, we held the number of relevant abstracts constant across prevalence conditions. Thus, prevalence was solely adjusted by modifying the number of irrelevant abstracts. Although this design controlled the confounding between prevalence and the number of relevant abstracts, it amplified the confounding between prevalence and the number of irrelevant abstracts. Therefore, we introduced an additional manipulation to examine how increasing the frequency of relevant and irrelevant abstracts—and consequently, the abstract collections size—affects the algorithm’s performance. To achieve prevalence rates of 1%, 2.5%, and 5% with a consistent frequency of 20 relevant abstracts, we sampled 2,000, 800, and 400 abstracts of the original abstract collections, respectively. When the frequency of relevant abstracts was set to 40, we sampled 4,000, 1,600, and 800 abstracts to achieve the corresponding prevalence rates. Introducing this frequency manipulation allowed us to evaluate the algorithm’s performance under two conditions: first, when prevalence was constant and the abstract collection size varied, and second, when the abstract collection size was constant at 800 and prevalence varied. We did not examine prevalences higher than 5%, as this would have resulted in too few abstracts to screen (e.g., 200 abstracts with only 20 being relevant).

### 5.2 Data

The criteria for selecting abstract collections in this study differed from those used in Study 1. While we also utilized the abstract collections initially gathered by König et al.^21^ for this study, our simulation design required the exclusion of any abstract collection containing fewer than 4,000 irrelevant and 50 relevant abstracts. As a result, nine abstract collections were eligible for inclusion from the domains of applied psychology (*n* = 1), clinical psychology (*n* = 1), developmental psychology (*n* = 4), and educational psychology (*n* = 3). The respective abstract collections are marked accordingly in Table 1. As described previously, we manipulated the prevalence and frequency of relevant abstracts. Relevant and irrelevant abstracts were sampled to match three prevalence conditions (1%, 2.5%, and 5%) and two frequency conditions (20 and 40 relevant abstracts). Each combination of conditions was replicated 1000 times, with each replication involving a random selection of abstracts from the original collections. This process resulted in a total of 9 abstract collections × 3 prevalence conditions × 2 frequency conditions × 1000 replications = 54,000 artificial abstract collections.

### 5.3 Simulation

Leveraging the Python API of ASReview,^39^ we conducted simulations for each of the 54,000 artificial abstract collections, using a method similar to König et al.^21^ These simulations were performed in R,^57^ utilizing the reticulate package^62^ to implement the Python code of ASReview’s Python API into the R environment. Given the superior performance of the LR + SBERT algorithm in Study 1, we exclusively used this algorithm to simulate AI-aided screening. All simulations adhered to ASReview’s default balancing strategy (i.e., dynamic resampling), alongside the certainty-based query strategy. In contrast to Study 1, we recalculated the inclusion probabilities for unseen abstracts after each additionally screened abstract, which is the default setting in ASReview. Moreover, this study introduced three different training set conditions: selecting 1, 2, or 5 relevant abstracts for training the algorithm. The number of irrelevant abstracts in each training set condition was set to 10. The training sets and screening set (abstracts for screening) varied across the 1000 replication runs due to the random sampling of abstracts. As a result, this approach led to a total of 162,000 simulation runs (54,000 artificial abstract collections × 3 training set sizes). Note that the training set was selected separately from the screening set. Thus, across all training set conditions, the same number of relevant and irrelevant abstracts was dedicated to the screening. In real-life screening situations, the training set is drawn from the total number of abstracts identified in the literature search. However, we decided to separate the training and screening sets to avoid confounding the training set effect with the number of relevant abstracts to detect.

### 5.4 Performance measures

The primary performance measure in this study was the SC (Eq. 2) at a sensitivity (see Eq. 1) of 95%. Notably, an SC in conditions with a frequency of 20 relevant abstracts reflected the SC when missing one relevant abstract, and with a frequency of 40, when missing two relevant abstracts. Moreover, to further explore the impact of the number of relevant abstracts included in the training set, we assessed the sensitivity at an SC of 10%. This metric, referred to as RFF@10%,^26^ measures the percentage of identified relevant studies after screening 10% of the abstracts.

### 5.5 Analysis

Mirroring the procedure from Study 1, our analysis is based on summary statistics including the mean, standard deviation, median, IQR, and the 25th, 75th, and 90th percentiles. We particularly focused on the median to compare performance across conditions, the IQR to assess variability, and the 90th percentile as an additional conservative performance measure. We visualized these metrics using bar plots. The bar plots delineated the main effects of prevalence, frequency, training set, and their interactions. To explore the distribution of the SC across various abstract collections, we visualized the data using violin plots. All analyses and visualizations were performed using the statistical software R,^57^ employing the dplyr package^58^ for calculations and the ggplot2 package^59^ for visualizations.

### 5.6 Results

Our analysis revealed a median of SC = 35.48% [19.95, 53.81] with a 90th percentile of 68.60%. This indicates that in 50% of all simulation runs, screening 35.70% of the abstracts was sufficient to identify 95% of the relevant abstracts, while 68.60% of the abstracts required screening to achieve the same identification rate in 90% of the simulation runs. Critically, performance varied across experimental conditions, with the median performance ranging from SC = 32% to SC = 40%, an IQR ranging from SC = 31% to SC = 37%, and a 90th percentile ranging from SC = 66% to SC = 71%.

**Figure 5:**
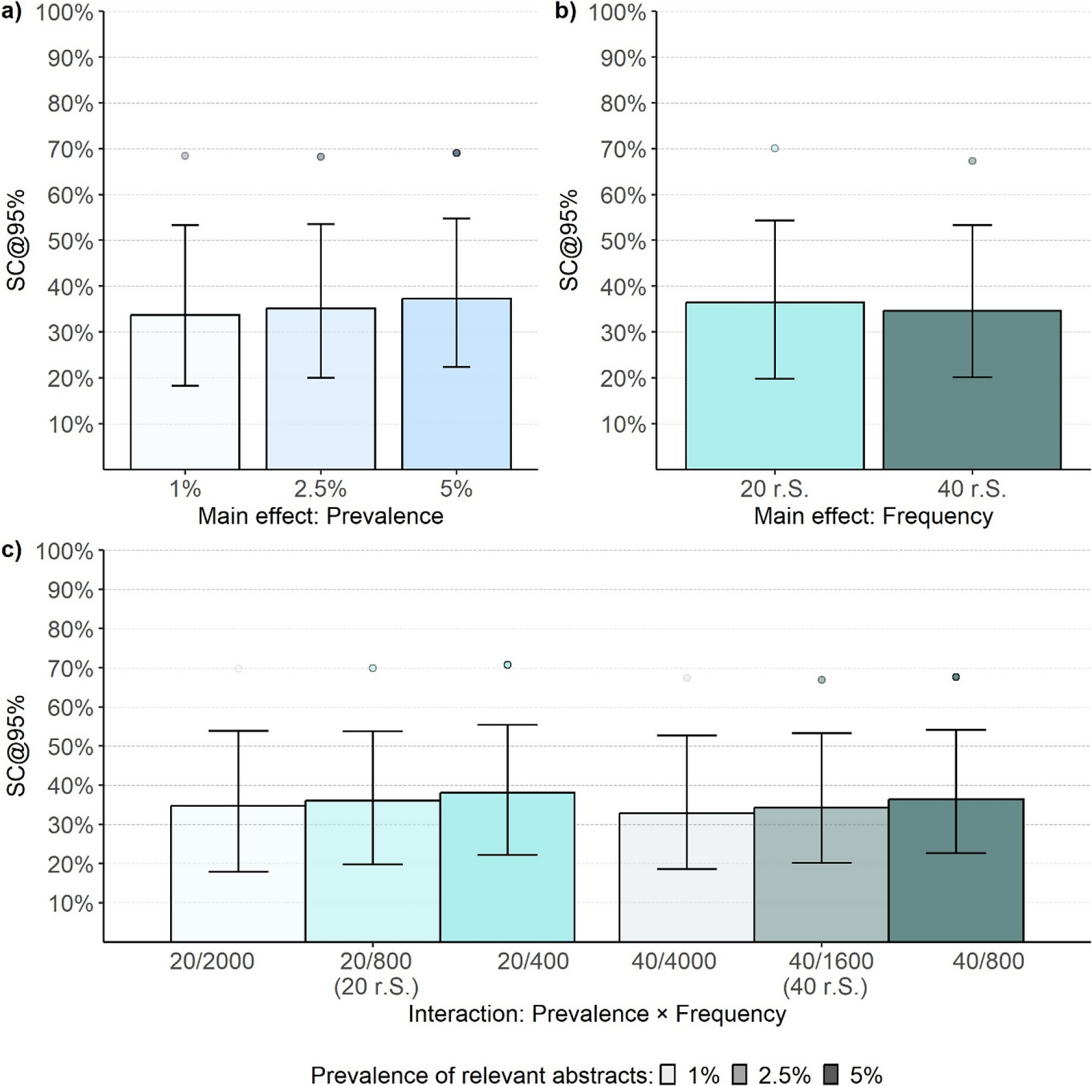
Screening cost at 95% sensitivity of the LR + SBERT algorithm for the main and interaction effects of prevalence and frequency (Study 2). *Note*: The bar plots reflect the median performance, the error bars represent the interquartile range, and the points represent the 90% percentile. Summary statistics for prevalence, frequency, and their interaction are based on 54000, 81,000, and 27,000 observations, respectively. r.S. = relevant abstracts in the screening set.

When evaluating the effect of prevalence, the ML algorithm performed best at a prevalence of 1% (SC *=* 33.74% [18.22, 53.27]). The performance declined by about 2% at a prevalence of 2.5% (SC *=* 35.12% [20.00, 53.54]) and again at a prevalence of 5% (SC = 37.26% [22.38, 54.76]). The variability in performance marginally decreased as prevalence increased (Figure 5a). However, a comparison of the 90th percentiles did not reveal differences between prevalence conditions (Table 3). Screening about 69% of the abstracts consistently resulted in a sensitivity of 95% in 90% of the simulation runs, regardless of prevalence. Notably, the effect of prevalence was confounded by the number of irrelevant abstracts, which was higher in low-prevalence conditions.

**Table 3:** Screening cost at 95% sensitivity by main effects (Study 2)

Main effect	Groups	*M*	*SD*	*Mdn*	*IQR*	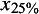	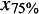	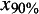
Prevalence	1%	36.10	22.36	33.74	35.05	18.22	53.27	68.44
		2.5%	37.10	21.60	35.12	33.54	20.00	53.54	68.23
		10%	38.99	20.96	37.26	32.38	22.38	54.76	69.05
	1 r.T.	37.97	21.88	36.10	34.13	20.43	54.55	69.41
Training set	2 r.T.	37.48	21.71	35.60	33.94	20.00	53.94	68.81
	5 r.T.	36.75	21.44	34.76	33.47	19.46	52.93	67.62
Frequency	20 r.S.	38.11	22.14	36.43	34.59	19.76	54.36	70.12
		40 r.S.	36.69	21.19	34.57	33.20	20.10	53.30	67.30

*Note:* Each data point regarding the effects of prevalence, training set, and sample size comprises 54,000, 54,000, and 81,000 data points, respectively. In the table cells, SC is represented as a percentage, yet the percent sign is omitted for clarity in presentation. r.S. = relevant abstracts in the screening set; r.T. = relevant abstracts in the training set; 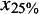 = 25% percentile; 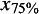 = 75% percentile; 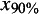 *=* 90% percentile.

Increasing the frequency of both relevant and irrelevant abstracts—and thereby expanding the abstract collection size—while maintaining the same prevalence led to a 2% improvement in relative performance for larger compared to smaller abstract collections (Figure 5b; Table 3).

An interaction of the frequency (abstract collection size) and prevalence was not observed. Across the three prevalence conditions (i.e., 1%, 2%, and 5%), a larger abstract collection size consistently led to a 2% improvement in performance. Moreover, at an abstract collection size of 800, increasing the prevalence of relevant abstracts from 2.5% (SC = 36.09% [19.76, 53.78]) to 5% (SC *=* 36.43% [22.62, 54.05]) did not result in a notably different median performance (Figure 5c; Table 4). Nonetheless, evaluating performance as FPR (Eq. 3) would have indicated slightly better performance in the 5% prevalence condition, as fewer irrelevant abstracts required screening in this scenario. This finding and the main effect of the abstract collection size contradict the main effect of prevalence.

**Table 4:** Screening cost at 95% sensitivity by sample size and prevalence (Study 2)

Frequency	Prevalence	*M*	*SD*	*Mdn*	*IQR*	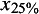	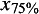	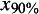
20 r.S.	1%	36.79	22.80	34.70	35.94	17.87	53.81	69.75
		2.5%	37.78	22.03	36.10	34.02	19.76	53.78	69.88
		5%	39.74	21.47	38.10	33.33	22.14	55.48	70.71
40 r.S.	1%	35.42	21.90	32.80	34.06	18.59	52.65	67.38
		2.5%	36.41	21.14	34.21	33.11	20.12	53.23	66.89
		5%	38.25	20.42	36.43	31.43	22.62	54.05	67.62

*Note:* Each data point consists of 27,000 data points. In the table cells, SC is represented as a percentage, yet the percent sign is omitted for clarity in presentation. r.S. = relevant abstracts in the screening set; 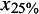 = 25% percentile; 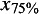 = 75% percentile; 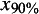 *=* 90% percentile.

**Figure 6:**
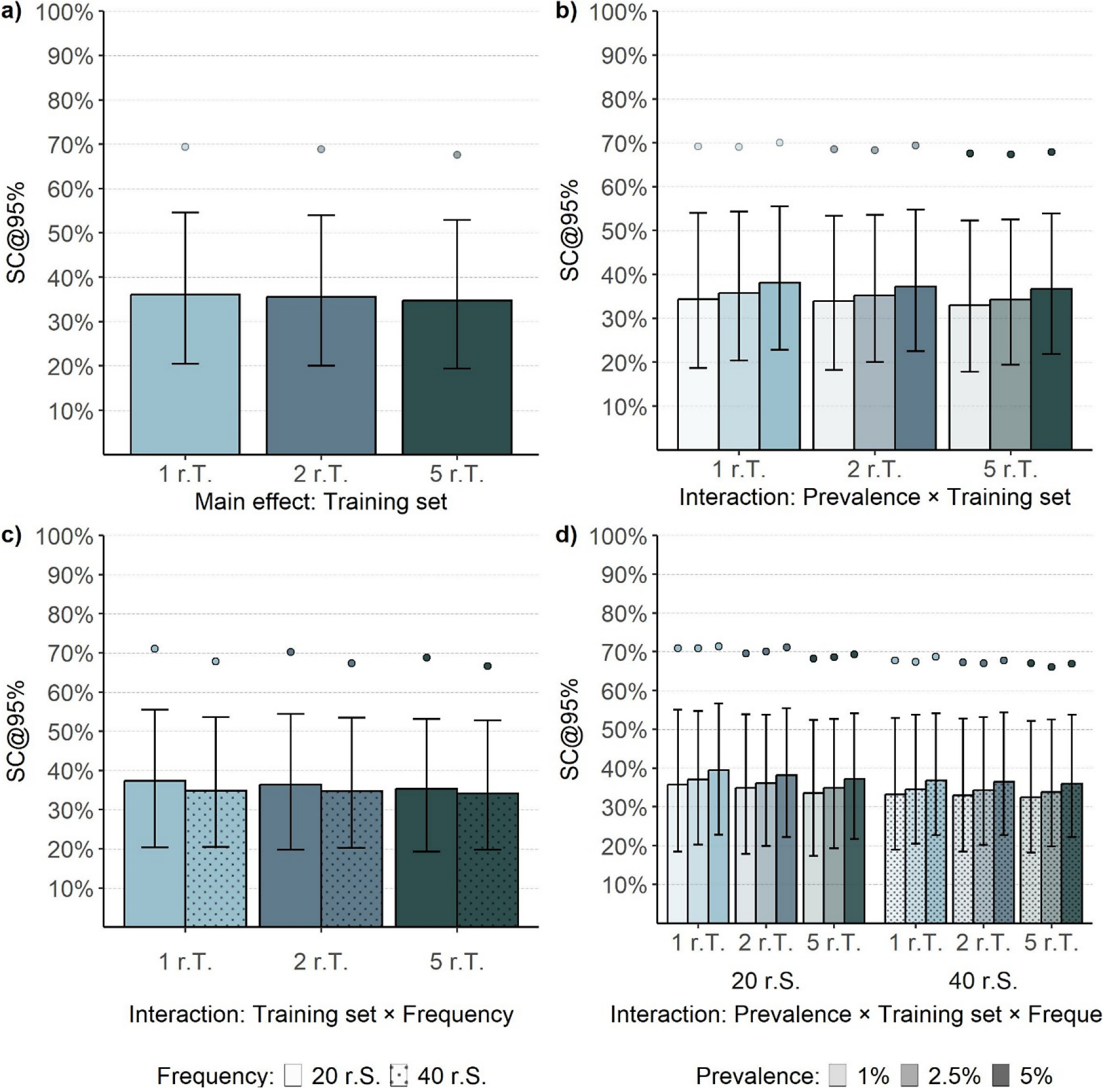
Screening cost at 95% sensitivity of the LR + SBERT algorithm for the main effect of training set and its interactions with prevalence and frequency (Study 2). *Note*: The bar plots reflect the median performance, the error bars represent the interquartile range, and the points represent the 90% percentile. Summary statistics are based on 54,000, 18,000, 27,000, and 9,000 observations for panels (a), (b), (c), and (d), respectively. r.S. = relevant abstracts in the screening set; r.T. = relevant abstracts in the training sets.

When examining the influence of the number of abstracts in the training set, only a marginal reduction in SC was observed (Figure 6a; Table 3). The median SC was only about 1% lower when five relevant abstracts were included in the training set (*SC* = 34.76% [19.46, 52.93]) compared to one relevant abstract (SC = 36.10% [20.43, 54.55]). Similarly, the increased training data also reduced the IQR by about 1%. However, when examining the interactions between the training set and other factors, we consistently observed a more pronounced but small effect of the training set in conditions with smaller frequencies of relevant and irrelevant abstracts (Figure 6b; see also Supplementary Table S4). Similarly, the interaction between the training set and the frequency of relevant and irrelevant abstracts showed a slightly stronger effect in conditions with smaller frequencies (Figure 6c; see also Supplementary Table S5). Furthermore, when examining the three-way interaction (Figure 6b), the influence of the training set was more substantial in conditions with the smallest abstract collection sizes (Figure 6d). For instance, the largest effect was observed in the condition with a 5% prevalence and a frequency of 20 relevant abstracts. However, the SC was only decreased by about 3% when five relevant abstracts were used (Supplementary Table S6). Notably, the evaluation of the algorithm’s performance after screening 10% of the abstracts (RFF@10) suggested that, at this stage of the screening process, a larger number of relevant abstracts in the training set improved sensitivity across all prevalence conditions (see Supplementary Figure S2).

Exploring the variability across and within abstract collections (Supplementary Figure S3) revealed notable differences in performance. The median SC varied widely between abstract collections, ranging from 7.86% to 66.91%. The average variability within abstract collections, however, was relatively stable across the collections varying between about 2% and 17% with an average of 10%. In contrast to the first study, this variability remained relatively consistent across prevalence conditions for all abstract collections.

### 5.7 Discussion of the results

Considering the results of this study, several interesting observations emerged, particularly when comparing the findings from Study 2 with those from Study 1. For instance, in this study, an increase in prevalence from 1% to 5% led to a rise in the SC by approximately 4%. Consequently, an additional 4% of the total abstracts needed to be screened to identify 95% of the relevant literature in higher prevalence conditions (RQ2.1), contrasting the effect of the first study. Notably, the manipulation design of this study resulted in fewer irrelevant abstracts in higher prevalence conditions. Therefore, when the SC is expressed as absolute numbers, fewer abstracts required screening in higher prevalence conditions. Additionally, the effect of prevalence has been confounded by the frequency of irrelevant abstracts. Indeed, comparing conditions with different prevalences of relevant abstracts (i.e., 2.5% and 5%) within abstract collections of the same overall size (i.e., 800 abstracts) revealed similar median performance, with less variability observed in the higher prevalence condition. However, the same median SC resulted in fewer irrelevant abstracts to screen when the percentage of relevant abstracts was higher. Consequently, performance increased slightly with an increase in prevalence when the overall abstract collection size was held constant, contradicting the effect observed from the prevalence manipulation. Moreover, while holding prevalence constant, conditions with larger frequencies of both relevant and irrelevant abstracts (abstract collections size) showed a 2% lower median SC (RQ2.2). Thus, the relative performance of the LR + SBERT algorithm increases with larger abstract collection sizes, consistent with correlational evidence from previous research.^22^

Our investigation into whether increasing the number of relevant abstracts in the training set reduces SCs and decreases variability in performance yielded unexpected results. While a clear improvement in performance was observed after screening 10% of the abstracts (RFF@10%), this enhancement was minimal when aiming for 95% sensitivity. Consequently, increasing the number of relevant abstracts to train the algorithm did not result in higher performance, although we observed a small effect when the abstract collection size was small (RQ2.3a). Furthermore, contrary to our expectations, a higher number of relevant abstracts in the training set did not result in a notably reduced variability in performance across simulation runs (RQ2.3b). These findings suggest that the algorithm was not biased notably by the specific characteristics of the abstracts used for training.

In summary, the effects observed in Study 2 were relatively small, indicating that the LR + SBERT algorithm is fairly robust against variations in prevalence, abstract collection size, and the number of relevant abstracts in the training set. Nevertheless, the relative performance appears to improve with an increase in abstract collection size. Aligning cutoff values for the time-based heuristic with the characteristics of the abstract collection could, therefore, enhance the efficiency of the screening progress. However, additional research is needed to derive specific recommendations for users across a variety of abstract collection sizes beyond those tested here.

## 6 General discussion

In this work, we evaluated 10 different ML algorithms available in the AI-aided screening tool ASReview.^44^ These algorithms prioritize abstracts based on their estimated probability of being relevant to the researcher screening them. We measured the SC for achieving 95% sensitivity (SC@95%). Thus, we evaluated performances in terms of the proportion of studies that needed to be screened until 95% of the relevant literature had been identified. To simulate AI-aided screening, we used abstracts from previously published meta-analyses and systematic reviews with meta-analysis. These abstract collections were originally compiled by König et al.,^21^ who examined the performance of stopping criteria in AI-assisted abstract screening. The dataset covered five psychological research domains: applied psychology, clinical psychology, developmental psychology, educational psychology, and social psychology. Although all domains fall within psychology, their diverse inclusion and exclusion criteria and interdisciplinary nature mirror features of other research fields, thereby offering the possibility that our findings generalize to some extent also to these fields. The substantial performance variations across abstract collections, ranging from 6% to 64% even for the best-performing algorithm (LR + SBERT), reflect patterns similar to those observed in other domains.^22^ ^,^ ^26^ ^,^ ^47^ Additionally, we explored how performance varied based on the prevalence of relevant abstracts and the overall size of the abstract collections, including both relevant and irrelevant abstracts. This manipulation builds on findings that reported correlations between these factors and ML algorithm performance.^22^ Moreover, we also examined the impact of the number of relevant abstracts used for training the ML algorithm to assess how variations influence algorithm performance. Understanding these factors provides valuable insights for users of AI-aided screening tools across research disciplines.

Consistent with previous findings,^22^ ^,^ ^43^ we observed that the ML algorithm combining the LR classifier with the SBERT feature extractor outperformed all other tested algorithms (Study 1). However, all algorithms exhibited considerable variability in performance, both across and within abstract collections. The within-collection variability arose mainly from drawing different samples of abstracts screened for the same meta-analyses and less from using different abstracts to train the algorithm. On average, the IQR within abstract collections was about 10%. The IQR of the median performance across abstract collections was considerably higher with about 20%. Similar notable variations in performance were observed within and across meta-analyses grouped by research domain (Study 1). However, the sample sizes for each domain are too small to draw definitive conclusions. Still, these variations emphasize the difficulty of estimating performance for specific abstract collections, even when relying solely on abstracts from the same domain. Notably, the variation observed in our study aligns with findings from evaluation studies that use abstract collections from different research fields.^22^ ^,^ ^26^ Because these studies did not manipulate data characteristics (i.e., prevalence of relevant abstracts and number of abstracts), our findings suggest that the observed variations stemmed primarily from differences in the data itself rather than other factors. Possible explanations include differences in inclusion and exclusion criteria or the strictness of term definitions. Vague terminology, or the use of multiple terms for the same concept, may confuse the algorithm and reduce its performance.^22^

Nonetheless, our experimental design revealed that the performance of all algorithms still depended on the prevalence of relevant abstracts, although this effect was ambiguous. When the number of relevant abstracts was held constant, and prevalence was manipulated by decreasing the number of irrelevant abstracts, higher prevalence resulted in a slight increase in SC (Study 2). Conversely, when prevalence was manipulated by increasing the number of relevant abstracts, the opposite pattern emerged (Study 1 and Study 2). Another observation was that higher frequencies of abstracts led to improved performance when prevalence was constant (Study 2). This finding also aligns with Campos et al.,^22^ who observed a positive correlation between the size of the abstract collection and the algorithm’s performance using unmanipulated data. One possible explanation for the relative performance improvement in terms of reduced SC could be the greater number of abstracts available for the algorithm to learn from when the total number of abstracts increases. However, the LR + SBERT algorithm exhibited somewhat similar performance across various conditions, with median SCs ranging from 32% to 40%, depending on the prevalence of relevant abstracts (Study 1 and Study 2) and the size of the abstract collection (Study 2). Nonetheless, to identify 95% of the relevant literature in 90% of AI-aided screening simulations, the LR + SBERT algorithm required screening between 58% and 71% of the abstracts (Study 1 and Study 2). This underscores the importance of aligning the decision on when to stop screening with the characteristics of the abstract collections (i.e., prevalence and frequency of abstracts) and the level of confidence desired in identifying at least 95% of the relevant literature.

Another aim of this study was to evaluate whether increasing the number of relevant abstracts in the training set affects the performance of the LR + SBERT algorithm. Some authors suggested that using abstracts known to be relevant prior to the literature search for training the algorithm could bias the algorithm’s ordering of abstracts.^45^ Accordingly, we proposed that increasing the number of randomly selected abstracts in the training set could reduce the influence of these characteristics, thereby enhancing algorithm performance and reducing variability. However, in our study, neither the median performance nor the variability in performance was notably influenced by increasing the number of relevant abstracts in the training set. A minor effect was observed only when the total number of abstracts to screen was low. Thus, using a single relevant abstract may be optimal for training the algorithm. This approach mitigates the risk associated with using multiple abstracts that share certain similarities. Moreover, it allows the additional abstracts to serve as a stopping criterion by using them as key studies, which must be identified during AI-aided screening before the process can be concluded.^45^

### 6.1 Limitations and future research directions

In our study, we explored factors impacting the performance of ML algorithms for AI-aided screening. However, our research has several limitations. First, while we manipulated the prevalence and frequency of relevant abstracts, along with the overall abstract collection size, our manipulation was not exhaustive. Future research could investigate additional prevalence rates and abstract collection sizes to better understand these effects and derive specific recommendations for users tailored to their needs.

Second, the abstract collections used to assess performance were all drawn from the field of psychology. Future research could replicate these findings in other disciplines. However, as demonstrated by the performance variations observed across abstract collections within and between psychological research domains, such variation may be more strongly related to the specificity of inclusion and exclusion criteria rather than the research field itself. Moreover, these variations persisted even after controlling for external characteristics of the abstract collections, such as prevalence and collection size. This observation aligns with findings suggesting that the performance of ML algorithms is influenced by the expertise of researchers conducting the screening, suggesting that performance largely depends on how consistently and accurately inclusion criteria are applied.^47^ Similarly, another study found that articles identified later in AI-assisted screening were frequently deemed irrelevant upon full-text review, suggesting that certain abstract characteristics may lead the algorithm to assign them a lower probability of relevance.^55^ Therefore, future research should explore how the specificity of inclusion criteria affects the performance of ML algorithms in AI-assisted screening.

As one of the reviewers correctly pointed out, our results cannot be generalized to systematic reviews that do not include meta-analyses. Some abstract collections in this study were derived from meta-analyses with systematic reviews (i.e., Alden et al.^63^; Karabinski et al.^64^; and Leijten et al.^65^), but most were from meta-analyses without systematic reviews. As systematic reviews do not require specific effect sizes, they often apply broader inclusion criteria. This might impact the algorithm performance negatively and result in a higher prevalence of relevant abstracts. With a higher prevalence, the proportion of relevant abstracts in a randomly selected subset (e.g., 5%) is higher, which directly influences the performance of the data-driven heuristics. In addition, broader inclusion criteria also increase the similarity between relevant and irrelevant abstracts, potentially diminishing the performance of ML algorithms. In such cases, the intervals between consecutive irrelevant abstracts are expected to be larger, which may also affect the performance of data-driven and time-based heuristics. Future research should further investigate how the type of research influences algorithm performance.

Third, we focused on the performance of specific ML algorithms provided by the screening tool ASReview. Prior research had shown that changing the ML algorithm during the screening process can enhance performance, although this effect did not appear when employing the LR + SBERT algorithm.^43^ Nonetheless, future research could investigate whether other algorithms and switching ML algorithms enhance effectiveness or reduce variability in performance. Moreover, in both of our studies, we utilized the default balancing and query strategies of ASReview.^39^ Investigating the impact of alternative strategies could lead to further optimization of AI-aided screening tools. In addition, while our results could be informative for users of other AI-aided screening tools, their generalizability is limited due to the different algorithms employed in these tools. Nonetheless, in many other screening tools, SVM algorithms are used and the performance for this algorithm documented in Table 2 might be informative for these tools. However, future research needs to replicate our results using other AI-aided screening tools.

Fourth, we measured performance only in terms of the proportion of abstracts screened (SC) and the proportion of the irrelevant abstracts screened. Considering other measures might have resulted in different findings. However, as our main study aim was to provide users with practical information regarding the screening process, we only included the SC measure. Nonetheless, interested readers can use this measure and information regarding the number of relevant abstracts to calculate other measures such as the WSS measure or to estimate the AUC (see Khalil et al.^16^).

Lastly, ML algorithms are inherently vulnerable to various biases.^66^ For instance, the representation bias can occur when the order of abstracts is influenced by non-random training data—a factor our simulation design successfully mitigated. In contrast, user interaction bias refers to the impact of misclassified data on the performance of the algorithm. As we did not manually screen the abstracts, we cannot rule out the possibility that this factor influenced our results.

### 6.2 Conclusions and practical recommendations

On the basis of our results, we provide recommendations for three key steps in the AI-aided screening process to enhance its sensitivity and efficiency: the random screening phase, stopping rule selection, and model setup. Notably, we incorporated suggestions from previous research,^21^ ^,^ ^22^ ^,^ ^45^ adapting them based on our findings. The final recommendations for each screening step are summarized in Table 5.

**Table 5:** Recommendations for the prescreening phase, stopping rule selection, and model setup

Screening phase	Recommendations
Random screening	*Random subset:* Randomly screen at least 1% of the abstracts, but no fewer than 100, until at least one relevant abstract is identified *Training set:* Select one relevant and one irrelevant abstract *Key studies:* Store the other identified relevant abstracts as key studies and mix them into the pool of unscreened abstracts *Prevalence estimate:* Estimate the prevalence of relevant abstracts based on their proportion in the subsample
Stopping rule selection	*Prevalence of 2.5% or lower:* Time-based heuristic: 40% Data-driven heuristic: 7.5% Key-study stopping rule: All key studies identified Breakout strategy: Time-based heuristic with 15% cutoff *Prevalence between 2.5% and 7.5%:* Time-based heuristic: 35% Data-driven heuristic: 5% Key-study stopping rule: All key studies identified Breakout strategy: Time-based heuristic with 10% cutoff *Prevalence above 7.5%* Time-based heuristic: 30% Data-driven heuristic: 2.5% Key-study stopping rule: All key studies identified Breakout strategy: Time-based heuristic with 5% cutoff
Model	*Classifier:* Logistic regression (LR) *Feature extractor:* Sentence-Bert (SBERT) *Query strategy:* Certainty-based (maximum) *balancing method:* Dynamic resampling (DR)

*Note:* These recommendations are based on the findings presented in this study, as well as research by Campos et al.,^22^ König et al.,^21^ and Boetje and van de Schoot.^45^

As outlined by Boetje and van de Schoot,^45^ the primary purpose of the random screening phase is to estimate the prevalence of relevant abstracts, compile a training set, and identify relevant articles to be used as key studies. To achieve this, we recommend randomly screening at least 1% of the abstracts retrieved through the literature search until at least one relevant abstract is identified. When 1% amounts to fewer than 100 abstracts, a minimum of 100 should be screened to enhance estimation accuracy. Prevalence can then be estimated by dividing the number of identified relevant abstracts by the total number of randomly screened abstracts and multiplying the quotient by the total number of articles retrieved in the literature search.^67^ Thereby, identified relevant abstracts can be used to compile a training set. However, in line with our findings, we propose constructing the training set by selecting only one relevant abstract and one irrelevant abstract. Any additional abstracts from known relevant articles, including those identified during random screening or prior to the literature search, should be designated as key studies and integrated with the unscreened abstracts. When employing AI-aided screening, the process should continue until all key studies have been identified. Nonetheless, because the key-study stopping rule primarily functions as a control mechanism, we recommend using it only in conjunction with other stopping rules. Similar to the SAFE method, we suggest combining the key-study stopping rule with both data-driven and time-based heuristics (see Table 5).

In a recent study, applying a 30% cutoff for the time-based heuristic and a 5% cutoff for the data-driven heuristic achieved a sensitivity of 95% across abstract collections with varying prevalence rates in education and educational psychology.^22^ However, because the authors examined performance without systematically manipulating prevalence or collection size, they were unable to provide specific recommendations for applying this stopping rule to datasets with different characteristics, as our findings suggest. Prior research on the data-driven heuristic has indeed shown performance differences across prevalence rates. König et al.^21^ observed that a 2.5% cutoff yielded sensitivities above 90% when prevalence was 5% or 10%, but only around 65% when prevalence was 1% or 0.5%.^21^ In consideration of this prior research, we recommend aligning cutoff values for the combined approach with prevalence estimates to enhance both sensitivity and efficiency. In addition, given that prevalence estimates derived from small subsets (e.g., 100 articles or 1% of total articles) may not be robust, we propose recommendations for broader prevalence categories such as below 2.5%, between 2.5% and 7.5%, and above 7.5% (see Table 5).

In our recommendations (see Table 5), we reduced the cutoff for the time-based heuristic for higher prevalence rates. Although higher prevalence generally increases SCs—given that the proportion of irrelevant abstracts remains similar, while the absolute number of relevant abstracts increases—our recommendation may appear arbitrary. However, the primary function of the time-based heuristic is to minimize the risk of premature stopping. When algorithm performance is limited, longer sequences of irrelevant abstracts are more likely to occur during the screening process, particularly under low-prevalence conditions. As the performance of the algorithms is typically less good in the beginning and toward the end of the AI-aided screening process, the data-driven heuristic bears the risk of being triggered too early. As more abstracts are added to the training set during screening, this scenario becomes less likely. Additionally, in higher prevalence conditions, the cutoff value for the data-driven heuristic can be lowered due to the greater proportion of relevant abstracts within the same span of abstracts. These findings justify lower cutoff values for both heuristics in higher prevalence conditions.

Another key feature of our recommendations is the implementation of a breakout stopping rule. Our findings revealed substantial variability in algorithm performance across different review datasets, even when factors such as the number and prevalence of relevant abstracts were held constant. This variability complicates the development of effective stopping strategies. As discussed above, our recommended combination of the data-driven and time-based heuristics is designed to enhance sensitivity, even when algorithm performance is suboptimal. However, when algorithm performance is high, a time-based heuristic may become unnecessary. To address this, we incorporated the breakout strategy—a data-driven heuristic with a high cutoff value. Although employing a high cutoff value may result in unnecessary workload when algorithm performance is average or poor, it can substantially reduce screening time in high-performing cases. For example, when the prevalence of relevant abstracts is approximately 1%, and all relevant abstracts are identified after screening 10% of the dataset, terminating the process upon encountering 15% consecutive irrelevant abstracts, rather than continuing until at least 40% of the dataset is screened, could considerably reduce the screening workload while ensuring a high identification rate of relevant articles.

For model selection, we recommend using the combination of the LR classifier and SBERT feature extractor as an ML algorithm for AI-aided screening in ASReview. This algorithm has demonstrated superior performance across various prevalence levels of relevant abstracts and exhibits the least variability across different abstract collections and training sets. However, while this algorithm performs optimally in combination with heuristic stopping rules, alternative models may be more suitable when employing non-heuristic stopping criteria.^21^ ^,^ ^22^ Additionally, we recommend using the default balancing and query strategy, as our results, along with the reviewed findings, are based on these settings.

To illustrate the recommendations described, consider the following example: A user retrieves 2,000 unique articles from a literature search and randomly screens 200 abstracts, identifying 6 relevant ones. Additionally, the user is aware of two relevant articles prior to the literature search. The user then estimates the prevalence and compiles the training set. In this case, the estimated prevalence is 3%. For the training set, one of the six relevant abstracts and one irrelevant abstract are randomly selected from the screened subset. The remaining five relevant abstracts from the random screening, along with the two known relevant abstracts, are marked as key studies and added to the 1,800 unscreened abstracts. As illustrated in Table 5, for a prevalence of 3%, screening should be stopped once 35% of the abstracts have been screened, all seven key studies have been identified, and 5% consecutive irrelevant abstracts have been detected. This approach could reduce the screening workload by 59%, accounting for 1% for random screening, 35% for AI-aided screening (time-based heuristic), and an additional 5% for AI-aided screening (data-driven heuristic). This translates to a reduction of about 1,180 abstracts, or approximately 10 h of screening time, assuming the user spends 30 s per abstract.^68^

Besides these recommendations, we strongly encourage users to adhere to state-of-the-art guidelines for AI-aided screening, which are designed to enhance the reproducibility and replicability of research syntheses.^69^ ^,^ ^70^ The findings from this study are expected to support researchers in improving both the efficiency and quality of their literature screening processes when using AI-aided tools such as ASReview. Additionally, these results may provide a foundation for future research aimed at improving the precision of performance estimates for ML algorithms. We also anticipate that our work will help foster greater trust in AI-aided screening tools, thereby encouraging their broader adoption in academic research.

## Supporting information

König et al. supplementary material König et al. supplementary material

## Data Availability

Study data and code can be found on the Open Science Framework: https://osf.io/53ter/.
