# Supplementary material for: Strategizing AI utilization for psychological literature screening: A comparative analysis of machine learning algorithms and key factors to consider
Source: Res Synth Methods. 2025 Dec 19;17(3):451–82. doi: 10.1017/rsm.2025.10053 (PMC13126213; doi:10.1017/rsm.2025.10053)
Supplement: König et al. supplementary material [file S1759287925100537sup001.docx]

Supplemental Material

**Strategizing AI Utilization for Psychological Literature Screening: A Comparative Analysis of Machine Learning Algorithms and Key Factors to Consider**

**Table S1**
*Research Topics of Meta-Analyses Used for Simulating AI-Aided Screening*

| Author (Year) | Category | Topic | Aim |
| --- | --- | --- | --- |
| Alden et al. (2021) | Applied Psychology | Digital Technology Use and Adolescent Mental Health | Analyzing the association between adolescents' digital technology use and mental health outcomes, focusing on identifying beneficial and harmful patterns of digital engagement. |
| Bottema-Beutel et al. (2021) | Developmental Psychology | Conflicts of Interest in Autism Research | Critically analyzing how often conflicts of interest occur in autism research and assessing their potential impact on intervention outcomes and study validity. |
| Bourke et al. (2022) | Developmental Psychology | Parental Mental Health and Child Behavior | Evaluating the effectiveness of interventions targeting parental mental health in improving behavioral and emotional outcomes in children. |
| Castro-Alonso et al. (2021) | Educational Psychology | Social-Emotional Learning and Academic Outcomes | Investigating whether social-emotional learning (SEL) interventions in educational settings significantly improve students' academic performance, emotional regulation, and social skills. |
| Cores et al. (2021) | Applied Psychology | Nutritional Interventions and Cognitive Development | Synthesizing research on nutritional interventions aimed at enhancing cognitive development and academic performance among children and identifying optimal dietary strategies. |
| Dailey & Bergelson (2022) | Developmental Psychology | Childhood Obesity Prevention | Evaluating interventions designed to prevent and manage obesity in childhood, assessing their effectiveness in achieving sustainable weight control and promoting healthy behaviors. |
| Endendijk et al. (2020) | Social Psychology | Teacher Development and Student Outcomes | Investigating the impact of professional development programs for teachers on student academic achievement, classroom management, and instructional quality. |
| Hall et al. (2023) | Educational Psychology | Reading Interventions | Conducting a comprehensive analysis of four decades of intervention research to determine the most effective approaches for improving reading abilities among elementary-aged children. |
| Hsieh et al. (2022) | Social Psychology | Exercise and Cognitive Function in Aging | Evaluating the impact of exercise and physical activity programs on cognitive functions such as memory, executive function, and attention in older adult populations. |
| Karabinski et al. (2021) | Applied Psychology | Peer Mentoring and Academic Success | Analyzing the impact of peer mentoring programs on college students' academic achievement, retention, social integration, and overall college experience. |
| Khazanov et al. (2022) | Clinical Psychology | Financial Incentives in Mental Health Treatments | Systematically evaluating the effectiveness of financial incentives as a method to increase patient adherence, participation, and outcomes in mental health treatment programs. |
| Leijten et al. (2021) | Clinical Psychology | Sleep Interventions and Academic Performance | Assessing the impact of sleep-focused interventions on academic performance and cognitive functioning among adolescents, exploring optimal intervention strategies and duration. |
| Liu et al. (2020) | Clinical Psychology | Stigma Towards Mental Illness | Synthesizing evidence on interventions aimed at reducing stigma toward mental illness, examining their effectiveness across diverse populations and intervention formats. |
| Ober et al. (2020) | Educational Psychology | Emotional Intelligence Training in the Workplace | Exploring the effectiveness of emotional intelligence training programs in improving workplace performance, interpersonal relationships, and organizational outcomes. |
| Reimer & Sengupta (2023) | Social Psychology | Ironic Effects of Social Integration | Synthesizing research evidence to understand the paradoxical negative outcomes (ironic effects) resulting from efforts aimed at promoting social integration and inclusion. |
| Schindler et al. (2023) | Social Psychology | Loneliness Interventions for the Elderly | Examining the effectiveness of targeted interventions designed to reduce loneliness among elderly populations, focusing on intervention types and their outcomes. |
| Simonsmeier et al. (2022) | Educational Psychology | Mindfulness Interventions and Sleep Quality | Assessing the effectiveness of mindfulness-based interventions in enhancing sleep quality and identifying specific aspects of mindfulness practices that yield the greatest improvements. |
| Tang et al. (2022) | Educational Psychology | Parental Involvement and Academic Achievement | Systematically reviewing research to evaluate how parental involvement influences academic achievement and identifying key involvement behaviors linked to positive educational outcomes. |
| Vermillet et al. (2022) | Developmental Psychology | Parental Influence on Infant Crying | Investigating the influence of different parental responses and caregiving behaviors on patterns and durations of infant crying during the first year of life. |
| Woods et al. (2022) | Applied Psychology | Gratitude Interventions and Psychological Health | Synthesizing existing evidence on gratitude interventions to determine their effects on psychological well-being, happiness, depression, and overall life satisfaction. |
| Zaneva et al. (2022) | Developmental Psychology | Virtual Reality for Anxiety Treatment | Assessing the clinical efficacy of virtual reality exposure therapy (VRET) as a treatment modality for various anxiety disorders, and identifying conditions under which it is most effective. |

*Note.* This table illustrates the variety of research topics and aims across the 21 meta-analyses from which screened abstract data were obtained.

**Table S2**

*Screening Cost at 95% Sensitivity by Machine Learning Algorithm (Study 1)*

| ML algorithm | *M* | *SD* | *Mdn* | *IQR* | $x_{25\%}$ | $x_{75\%}$ | $x_{90\%}$ |
| --- | --- | --- | --- | --- | --- | --- | --- |
| LR+SBERT | 36.78 | 18.61 | 35.28 | 24.74 | 23.42 | 48.16 | 61.84 |
| LR+TFIDF | 41.95 | 19.38 | 39.02 | 27.41 | 28.18 | 55.58 | 68.17 |
| nn2layer+SBERT | 42.27 | 20.60 | 39.95 | 29.89 | 27.44 | 57.33 | 70.61 |
| NB+TFIDF | 42.85 | 18.98 | 41.49 | 27.43 | 28.80 | 56.23 | 67.40 |
| LR+doc2vec | 43.44 | 17.86 | 42.03 | 25.13 | 30.83 | 55.96 | 66.58 |
| RF+doc2vec | 47.04 | 19.70 | 45.50 | 27.27 | 33.07 | 60.35 | 74.03 |
| SVM+TFIDF | 47.34 | 21.22 | 44.52 | 31.17 | 31.98 | 63.15 | 77.29 |
| nn2layer+doc2ve | 47.73 | 20.03 | 46.07 | 27.10 | 33.71 | 60.81 | 75.06 |
| RF+TFIDF | 50.14 | 19.38 | 48.85 | 27.11 | 36.48 | 63.60 | 76.36 |
| RF+SBERT | 39.69 | 19.96 | 37.50 | 27.26 | 25.86 | 53.12 | 67.27 |

*Note:* For each machine learning algorithm, descriptive statistics are based on 84,000 observations. In the table cells, Screening Cost is represented as a percentage, yet the percent sign is omitted for clarity in presentation. $x_{25\%}$ = 25% percentile; $x_{75\%}$= 75% percentile; $x_{90\%}$ *=* 90% percentile

**Table S3**

*Screening Cost at 95% Sensitivity by Prevalence Ratio (Study 1)*

| Prevalence | *M* | *SD* | *Mdn* | *IQR* | $x_{25\%}$ | $x_{75\%}$ | $x_{90\%}$ |
| --- | --- | --- | --- | --- | --- | --- | --- |
| 0.5% | 49.10 | 24.37 | 48.33 | 38.48 | 29.75 | 68.23 | 82.88 |
| 1% | 44.25 | 22.04 | 42.45 | 33.39 | 27.50 | 60.88 | 74.93 |
| 5% | 40.50 | 16.53 | 39.23 | 24.61 | 28.28 | 52.89 | 62.77 |
| 10% | 41.85 | 14.16 | 40.92 | 19.96 | 32.12 | 52.07 | 61.63 |

*Note:* Each data point consists of 210,000 observations. In the table cells, Screening Cost is represented as a percentage, yet the percent sign is omitted for clarity in presentation. $x_{25\%}$ = 25% percentile; $x_{75\%}$= 75% percentile; $x_{90\%}$ *=* 90% percentile. $x_{25\%}$ = 25% percentile; $x_{75\%}$= 75% percentile; $x_{90\%}$ *=* 90% percentile

**Table S4**

*Screening Cost at 95% Sensitivity by Prevalence and Training Set (Study 2)*

| Prevalence | Training set | *M* | *SD* | *Mdn* | *IQR* | $x_{25\%}$ | $x_{75\%}$ | $x_{90\%}$ |
| --- | --- | --- | --- | --- | --- | --- | --- | --- |
| 1% | 1 r.T. | 36.70 | 22.58 | 34.38 | 35.32 | 18.66 | 53.99 | 69.16 |
|  | 2 r.T. | 36.16 | 22.35 | 33.96 | 35.15 | 18.26 | 53.42 | 68.54 |
|  | 5 r.T. | 35.46 | 22.14 | 32.97 | 34.51 | 17.77 | 52.28 | 67.60 |
|  |  |  |  |  |  |  |  |  |
| 2.5% | 1 r.T. | 37.67 | 21.79 | 35.73 | 33.98 | 20.37 | 54.34 | 69.09 |
|  | 2 r.T. | 37.17 | 21.62 | 35.24 | 33.48 | 20.06 | 53.54 | 68.35 |
|  | 5 r.T. | 36.46 | 21.37 | 34.30 | 33.13 | 19.44 | 52.56 | 67.32 |
|  |  |  |  |  |  |  |  |  |
| 5% | 1 r.T. | 39.53 | 21.15 | 38.10 | 32.62 | 22.86 | 55.48 | 70.00 |
|  | 2 r.T. | 39.12 | 21.04 | 37.26 | 32.26 | 22.50 | 54.76 | 69.40 |
|  | 5 r.T. | 38.34 | 20.69 | 36.67 | 32.02 | 21.90 | 53.93 | 67.86 |

*Note:* Each data point consists of 18,000 data points. In the table cells, Screening Cost is represented as a percentage, yet the percent sign is omitted for clarity in presentation. r.T. = Relevant abstracts in the training set; $x_{25\%}$ = 25% percentile; $x_{75\%}$= 75% percentile; $x_{90\%}$ *=* 90% percentile

**Table S5**

*Screening Cost at 95% Sensitivity by Sample Size and Training Set (Study 2)*

| Frequency | Training set | *M* | *SD* | *Mdn* | *IQR* | $x_{25\%}$ | $x_{75\%}$ | $x_{90\%}$ |
| --- | --- | --- | --- | --- | --- | --- | --- | --- |
| 20 r.S. | 1 r.T. | 38.94 | 22.40 | 37.38 | 35.10 | 20.40 | 55.50 | 71.10 |
|  | 2 r.T. | 38.19 | 22.20 | 36.43 | 34.63 | 19.76 | 54.39 | 70.25 |
|  | 5 r.T. | 37.19 | 21.79 | 35.37 | 33.83 | 19.27 | 53.10 | 68.81 |
|  |  |  |  |  |  |  |  |  |
| 40 r.S. | 1 r.T. | 36.99 | 21.31 | 34.88 | 33.17 | 20.43 | 53.60 | 67.87 |
|  | 2 r.T. | 36.78 | 21.19 | 34.65 | 33.27 | 20.18 | 53.45 | 67.32 |
|  | 5 r.T. | 36.31 | 21.08 | 34.16 | 33.08 | 19.72 | 52.80 | 66.71 |

*Note:* Each data point consists of 27,000 data points. In the table cells, Screening Cost is represented as a percentage, yet the percent sign is omitted for clarity in presentation. r.T. = relevant abstracts in the training set; r.S. = relevant abstracts in the screening set; $x_{25\%}$ = 25% percentile; $x_{75\%}$= 75% percentile; $x_{90\%}$ *=* 90% percentile

**Table S6**

*Screening Cost at 95% Sensitivity by Sample Size, Prevalence, and Training Set (Study 2)*

| Frequency | Prevalence | Training set | *M* | *SD* | *Mdn* | *IQR* | $x_{25\%}$ | $x_{75\%}$ | $x_{90\%}$ |
| --- | --- | --- | --- | --- | --- | --- | --- | --- | --- |
| 20 r.S. | 1% | 1 r.T. | 37.72 | 23.15 | 35.79 | 36.65 | 18.42 | 55.06 | 70.94 |
|  |  | 2 r.T. | 36.82 | 22.78 | 34.85 | 36.00 | 17.91 | 53.91 | 69.60 |
|  |  | 5 r.T. | 35.85 | 22.42 | 33.51 | 35.06 | 17.38 | 52.44 | 68.22 |
|  |  |  |  |  |  |  |  |  |  |
|  | 2.5% | 1 r.T. | 38.58 | 22.26 | 37.07 | 34.51 | 20.24 | 54.76 | 70.85 |
|  |  | 2 r.T. | 37.86 | 22.06 | 36.10 | 33.90 | 19.88 | 53.78 | 70.00 |
|  |  | 5 r.T. | 36.91 | 21.75 | 34.88 | 33.41 | 19.27 | 52.68 | 68.55 |
|  |  |  |  |  |  |  |  |  |  |
|  | 5% | 1 r.T. | 40.52 | 21.67 | 39.52 | 33.81 | 22.86 | 56.67 | 71.43 |
|  |  | 2 r.T. | 39.89 | 21.62 | 38.10 | 33.33 | 22.14 | 55.48 | 71.19 |
|  |  | 5 r.T. | 38.81 | 21.08 | 37.14 | 32.38 | 21.67 | 54.05 | 69.29 |
|  |  |  |  |  |  |  |  |  |  |
| 40 r.S. | 1% | 1 r.T. | 35.68 | 21.96 | 33.16 | 33.99 | 18.95 | 52.95 | 67.72 |
|  |  | 2 r.T. | 35.50 | 21.89 | 32.95 | 34.23 | 18.51 | 52.75 | 67.33 |
|  |  | 5 r.T. | 35.07 | 21.84 | 32.43 | 33.87 | 18.24 | 52.11 | 67.08 |
|  |  |  |  |  |  |  |  |  |  |
|  | 2.5% | 1 r.T. | 36.76 | 21.28 | 34.51 | 33.23 | 20.49 | 53.72 | 67.38 |
|  |  | 2 r.T. | 36.48 | 21.15 | 34.33 | 32.99 | 20.18 | 53.17 | 67.02 |
|  |  | 5 r.T. | 36.00 | 20.98 | 33.84 | 32.74 | 19.76 | 52.50 | 66.10 |
|  |  |  |  |  |  |  |  |  |  |
|  | 5% | 1 r.T. | 38.55 | 20.57 | 36.79 | 31.31 | 22.74 | 54.05 | 68.69 |
|  |  | 2 r.T. | 38.34 | 20.41 | 36.43 | 31.55 | 22.74 | 54.29 | 67.74 |
|  |  | 5 r.T. | 37.86 | 20.28 | 35.95 | 31.43 | 22.26 | 53.69 | 66.90 |
|  |  |  |  |  |  |  |  |  |  |

*Note:* Each data point consists of 9,000 data points. In the table cells, Screening Cost is represented as a percentage, yet the percent sign is omitted for clarity in presentation. Training set reflects the number of relevant abstracts used for training. r.S. = relevant abstracts in the screening set; r.T. = relevant abstracts in the training set; $x_{25\%}$ = 25% percentile; $x_{75\%}$= 75% percentile; $x_{90\%}$ *=* 90% percentile

**Figure S1**

*Violin Plot Pertaining the Distribution of Screening Cost at 95% Sensitivity Across Conditions and Abstract Collections for the LR+SBERT algorithm (Study 1)*

*
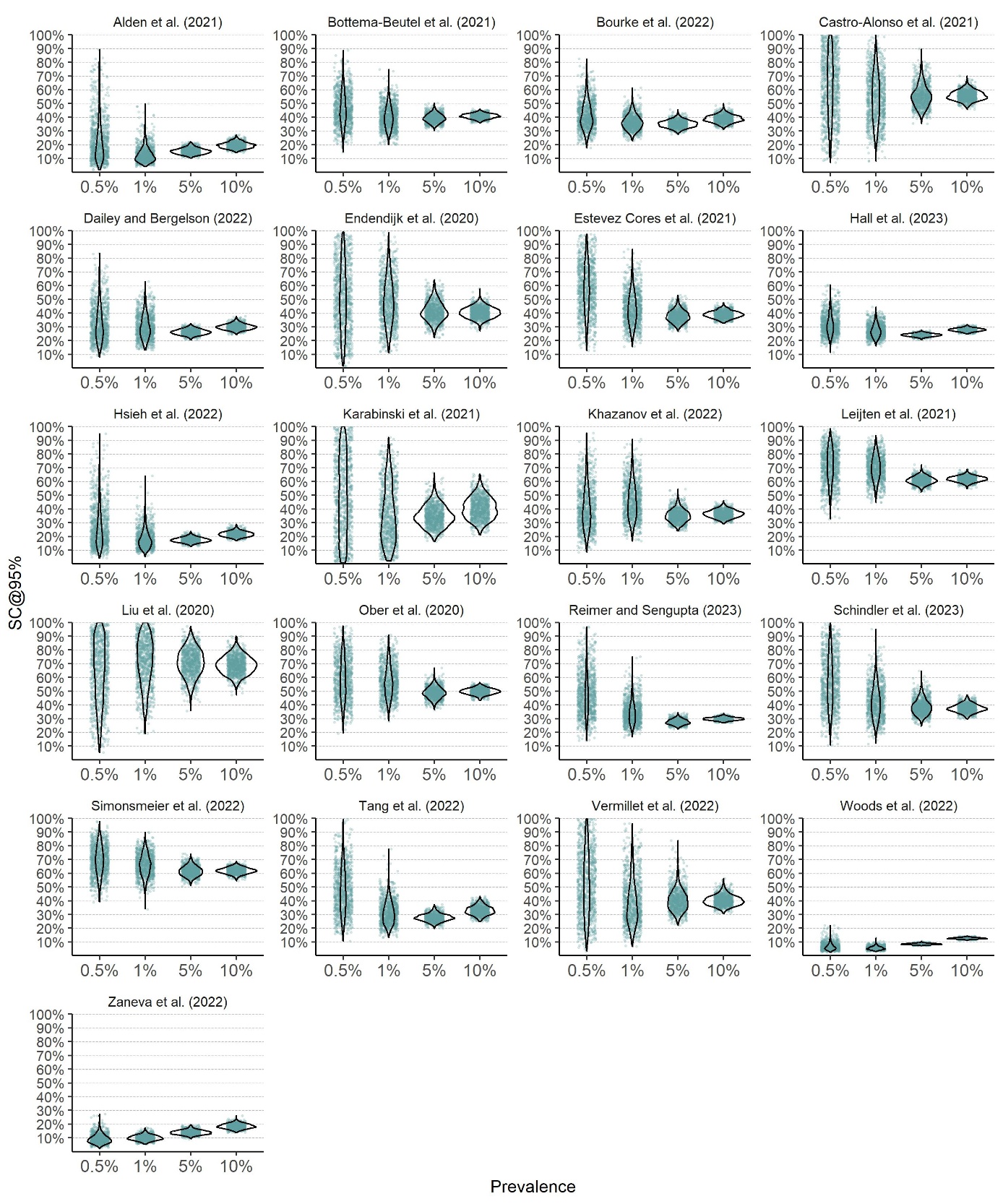
*

*Note.* Each point reflects the Screening Cost for a single simulation run.

**Figure S2**

*Bar Plot Pertaining the Sensitivity after Screening 10% (RFF@10%) of the Abstracts (Study 2)*


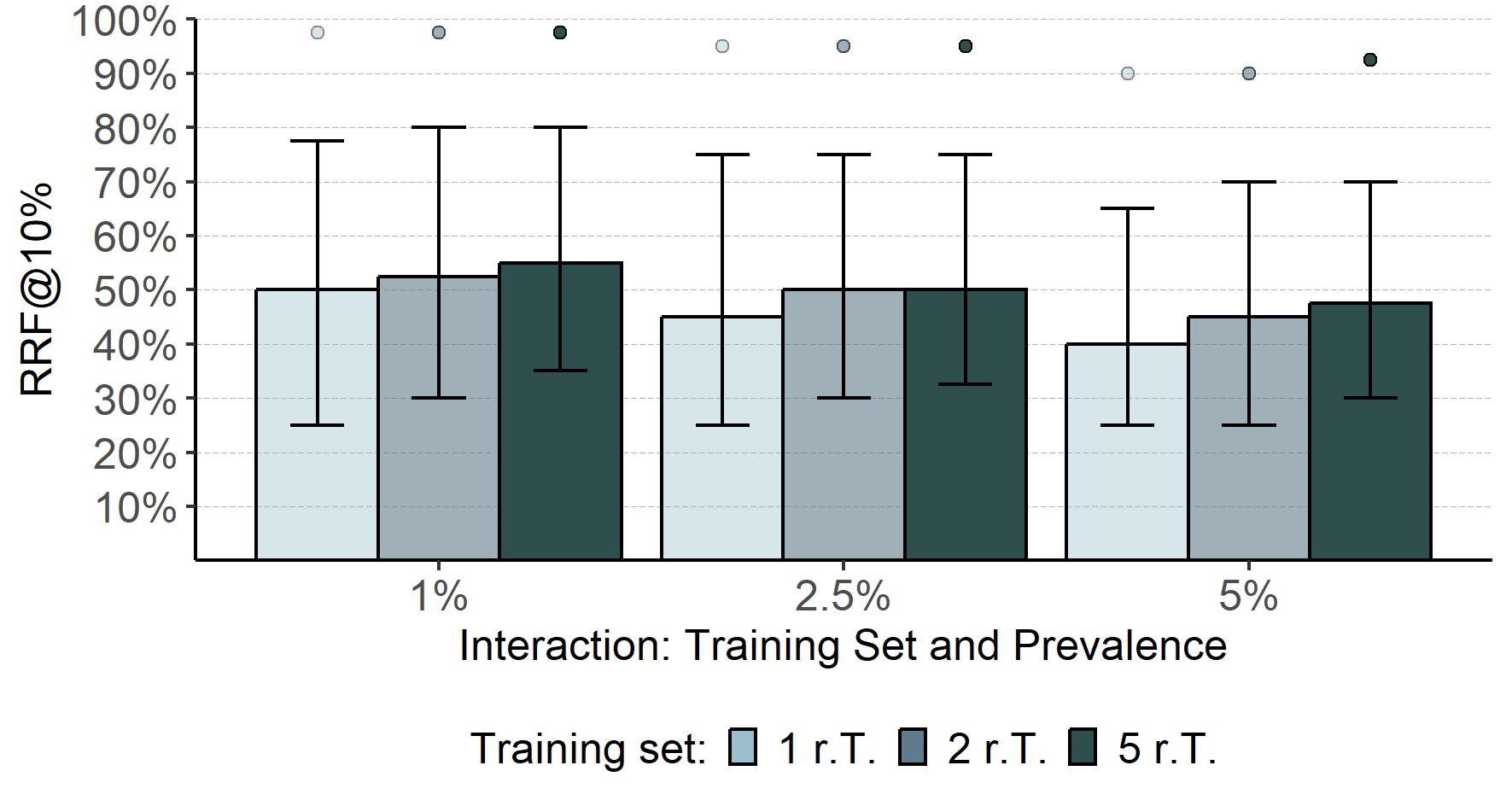


*Note.* The bar-plots reflect the median performance, the error bars represent the interquartile range, and the points the 90% percentile. Each summary statistic summarizes 18,000 observations. r.T. = relevant abstracts in the training sets. RRF@10% = percentage of the Relevant Records Found (RRF) after screening 10% of the total.

**Figure S3**

*Violin Plots Showing the Distribution of Screening Cost at a 95% Sensitivity* *(SC@95%) for the LR+SBERT algorithm Across Prevalence Conditions (1%, 2.5%, 5%) within Abstract* *Collections (Study 2)*

*
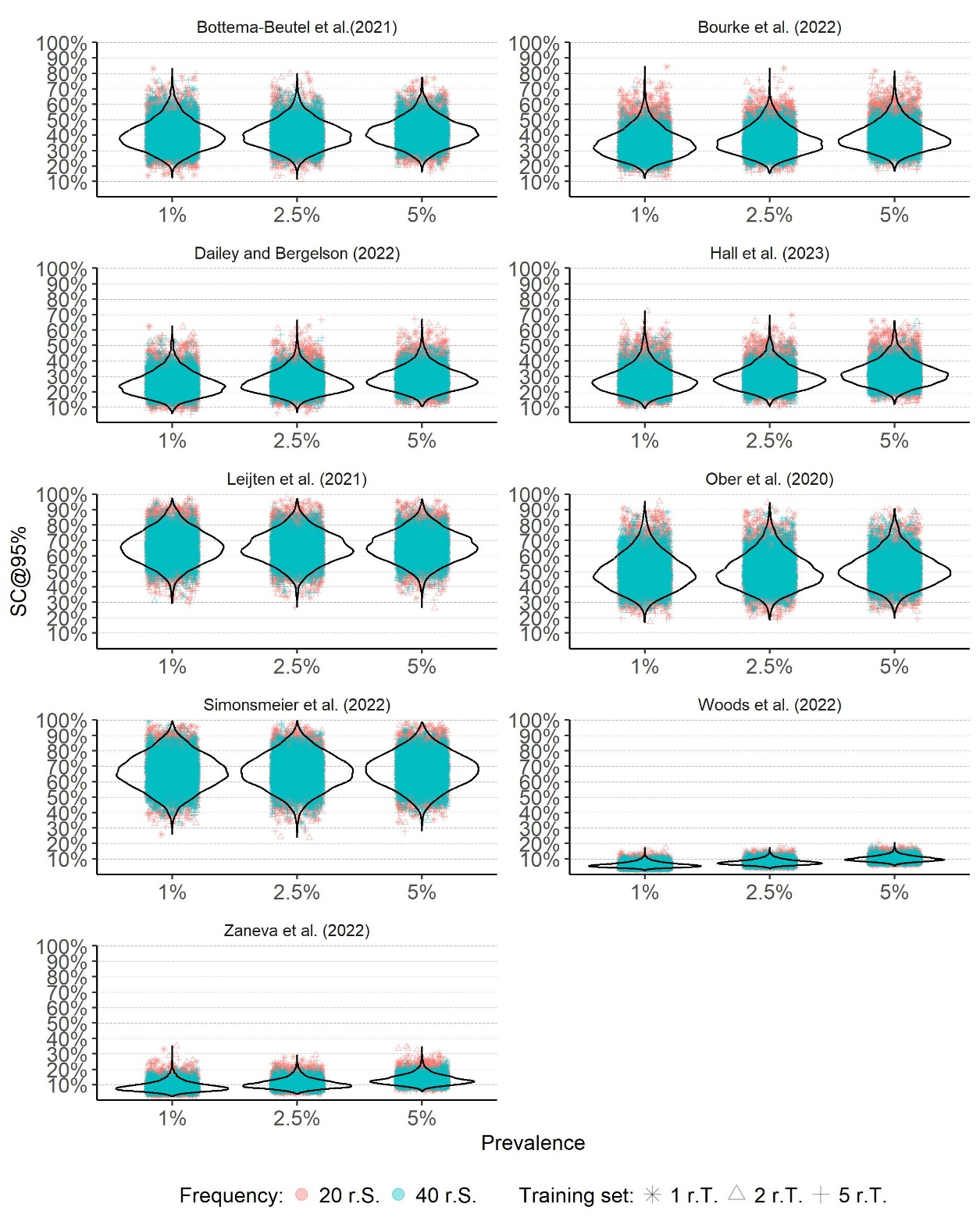
*

*Note.* Each point reflects the Screening Cost for a single simulation run.
